# In situ tumour arrays reveal early environmental control of cancer immunity

**DOI:** 10.1038/s41586-023-06132-2

**Published:** 2023-05-31

**Authors:** Guadalupe Ortiz-Muñoz, Markus Brown, Catherine B. Carbone, Ximo Pechuan-Jorge, Vincent Rouilly, Henrik Lindberg, Alex T. Ritter, Gautham Raghupathi, Qianbo Sun, Tess Nicotra, Shreya R. Mantri, Angela Yang, Jonas Doerr, Deepti Nagarkar, Spyros Darmanis, Benjamin Haley, Sanjeev Mariathasan, Yulei Wang, Carlos Gomez-Roca, Carlos Eduardo de Andrea, David Spigel, Thomas Wu, Lelia Delamarre, Johannes Schöneberg, Zora Modrusan, Richard Price, Shannon J. Turley, Ira Mellman, Christine Moussion

**Affiliations:** 1 Genentech, South San Francisco, CA USA; 2 Department of Computer Science, Stanford University, Stanford, CA USA; 3 IUCT, Institut Universitaire du Cancer de Toulouse, Toulouse, France; 4 Department of Pathology, Clínica Universidad de Navarra, Pamplona, Spain; 5 Sarah Cannon Research Institute, Nashville, TN USA; 6 Department of Pharmacology, UCSD, San Diego, CA USA; 7 Department of Chemistry & Biochemistry, UCSD, San Diego, CA USA

**Keywords:** Cancer microenvironment, Cancer models, Cellular immunity, Imaging the immune system, Tumour immunology

## Abstract

The immune phenotype of a tumour is a key predictor of its response to immunotherapy^1–4^. Patients who respond to checkpoint blockade generally present with immune-inflamed^5–7^ tumours that are highly infiltrated by T cells. However, not all inflamed tumours respond to therapy, and even lower response rates occur among tumours that lack T cells (immune desert) or that spatially exclude T cells to the periphery of the tumour lesion (immune excluded)^8^. Despite the importance of these tumour immune phenotypes in patients, little is known about their development, heterogeneity or dynamics owing to the technical difficulty of tracking these features in situ. Here we introduce skin tumour array by microporation (STAMP)—a preclinical approach that combines high-throughput time-lapse imaging with next-generation sequencing of tumour arrays. Using STAMP, we followed the development of thousands of arrayed tumours in vivo to show that tumour immune phenotypes and outcomes vary between adjacent tumours and are controlled by local factors within the tumour microenvironment. Particularly, the recruitment of T cells by fibroblasts and monocytes into the tumour core was supportive of T cell cytotoxic activity and tumour rejection. Tumour immune phenotypes were dynamic over time and an early conversion to an immune-inflamed phenotype was predictive of spontaneous or therapy-induced tumour rejection. Thus, STAMP captures the dynamic relationships of the spatial, cellular and molecular components of tumour rejection and has the potential to translate therapeutic concepts into successful clinical strategies.

## Abstract (web summary)

Skin tumour array by microporation (STAMP) captures the dynamic relationships of spatial, cellular and molecular components of tumour rejection and has the potential to translate therapeutic concepts into successful clinical strategies.

## Main

**Fig. 1:**
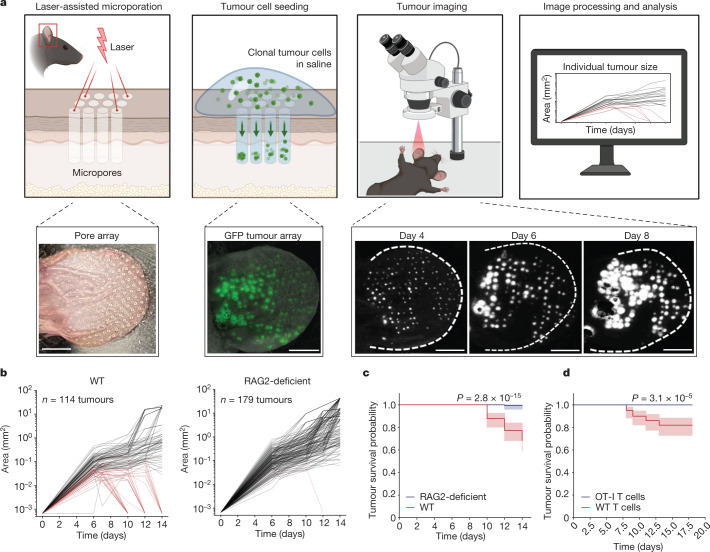
STAMP reveals local T-cell-mediated rejection of clonal skin tumour array. **a**, The STAMP workflow. Skin microporation using the P.L.E.A.S.E. laser device and subsequent seeding of tumour arrays from cell suspension. Individual tumours of the array are longitudinally tracked using epifluorescence microscopy and the growth kinetics are analysed by automated computation. Scale bars, 4 mm. **b**, Automated analysis of the growth kinetics of individual KPP-eGFP tumours (area in mm^2^) inside the same tumour array implanted into RAG2-deficient (*n* = 179 tumours, *n* = 4 mice) or wild-type (WT) (*n* = 114 tumours, *n* = 3 mice) mice. Sampling is representative of *n* = 5 mice per group. The red lines indicate tumours that were rejected, and the grey lines indicate tumours that persist. **c**, The survival probability of individual tumours of KPP-eGFP arrays as described in **b**. The centre line shows the Kaplan–Meier curve and the shaded area shows the 95% confidence interval. Statistical analysis was performed using a log-rank test. **d**, The survival probability of individual tumours of KPP-eGFP arrays implanted into RAG2-deficient mice reconstituted by adoptive transfer of tdTomato^+^ T cells from either WT mice (*n* = 100 tumours, *n* = 2 mice) or OT-I mice (*n* = 88 tumours, *n* = 2 mice). Sampling is representative of *n* = 2 independent experiments, *n* = 7 mice per group. The centre line shows the Kaplan–Meier curve and the shaded area shows the 95% confidence interval. Statistical analysis was performed using log-rank tests. Source Data

**Extended Data Fig. 1:**
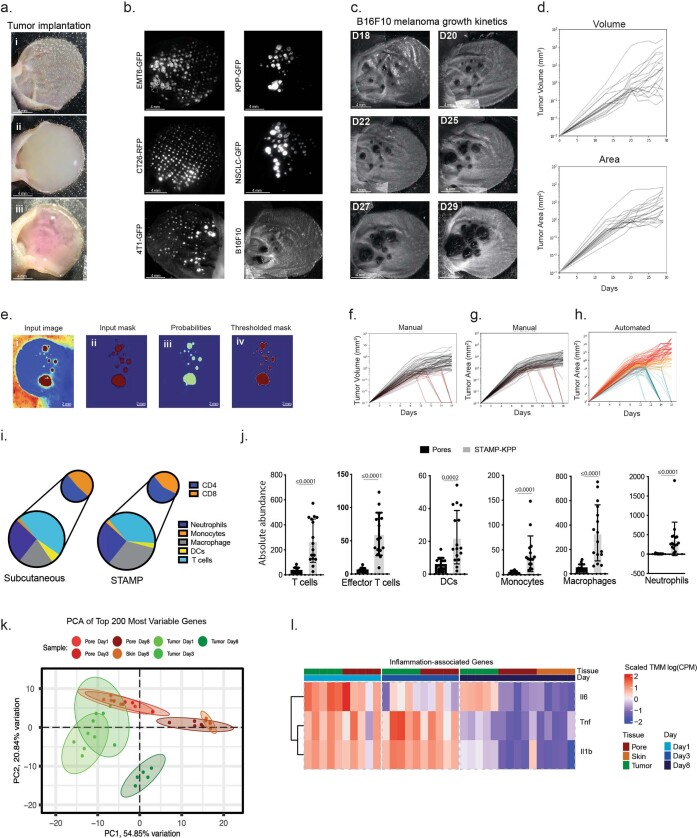
Implantation, inflammatory response, and growth analysis of STAMP microtumours. **a**, Image of **(i)** ears after P.L.E.A.S.E.® Laser microporation, **(ii)** microporated ears covered with tumour cell suspension, **(iii)** microporated ear seeded with tumour cells and covered with Matrigel. **b**, Representative tumour arrays of different tumour cell lines, n ≧ 10 animals per group. **c**, Representative time course image series of orthotopic B16F10 model cell line growing in STAMP. n = 5 animals. **d**, Manual analysis of growth kinetics of individual B16F10 STAMP tumours using ellipsoid formula to calculate volumes over time (upper panel) and using tumour segmentation to calculate area over time (lower panel). n = 27 tumours, 2 animals pooled. **e**, Representative tumour segmentation performed on validation images. (i) Input images, (ii) manually generated classification mask, (iii) features extracted after the penultimate upsampling step, (iv) output segmentation mask. **f-h**, Validation of the high content image analysis pipeline. **f**, Manual analysis of growth kinetics of individual KPP-EGFP tumour volumes (mm^3^) using the ellipsoid formula. **g**, Manual analysis of growth kinetics of individual tumour areas (mm^2^) using tumour segmentation. **h**, Automated analysis of growth kinetics of individual tumour areas (mm^2^), **f-h**, n = 72 tumours, 3 animals pooled. **i**, Lymphoid and myeloid immune cell profiling of STAMP tumour array and subcutaneous tumours of KPP-EGFP cancer cells 18 days post tumour implantation and pooled. n = 6 ears, n = 3 mice for STAMP. n = 5 mice for subcutaneous tumours. **j**, Lymphoid and myeloid immune cell profiling of PBS or KPP-EGFP-seeded micropores. STAMP tumour arrays were harvested 18 days post tumour implantation and pooled by ear. n = 16 ears, n = 8 mice. Data are mean +/− s.d. Statistical analysis was performed using a two-tailed Mann-Whitney U-test. *P* values are shown in the graph. **k**, Principal Component Analysis (PCA) of the 200 most variable genes of the RNA-seq data from healthy skin, pores and KPP-EGFP microtumours biopsied at day 1, day 3 and day 8 after tumour seeding and/or laser poration. Samples are coloured according to day and experimental group (tumour, pores and healthy skin). Ellipses at 95% confidence level are shown for each group. The percentage of explained variance for each principal component is annotated on the principal component axes. **j**, Heatmap of the z-scored gene expression values for Tnf, Il6, Il1b. Columns are annotated as in **k** and rows are hierarchically clustered to show genes with similar patterns of expression across the samples. Source Data

The STAMP technique uses an infrared laser^9^ to create an array of hundreds of pores in the dermis of the mouse ear. Tumour cells expressing a fluorescent reporter are seeded into each pore and tumour growth is monitored over the next 4–6 weeks using live fluorescence microscopy (Fig. 1a and Extended Data Fig. 1a–d). To track and measure the growth of dozens of individual microtumours in a single array, we developed a computational pipeline using a convolutional neural network (Extended Data Fig. 1e–h). STAMP can successfully implant orthotopic (melanoma) or heterotopic (mammary, pancreas, lung and colon carcinoma) tumour cell lines in the skin.

## Local T cell-mediated tumour rejection

To characterize immune infiltration into STAMP tumours, we compared a clonal KPP-eGFP cell line (pancreatic ductal adenocarcinoma)^10^ injected subcutaneously into the flank or using STAMP on the ear. We observed similar immune infiltrates at 10 days after tumour implantation, regardless of the implantation method (Extended Data Fig. 1i). Importantly, immune cell profiling using flow cytometry demonstrated a specific tumour-dependent recruitment of lymphoid and myeloid populations (Extended Data Fig. 1j), and the inflammation associated with laser microporation completely resolved a few days after tumour implantation (Extended Data Fig. 1k–l).

**Extended Data Fig. 2:**
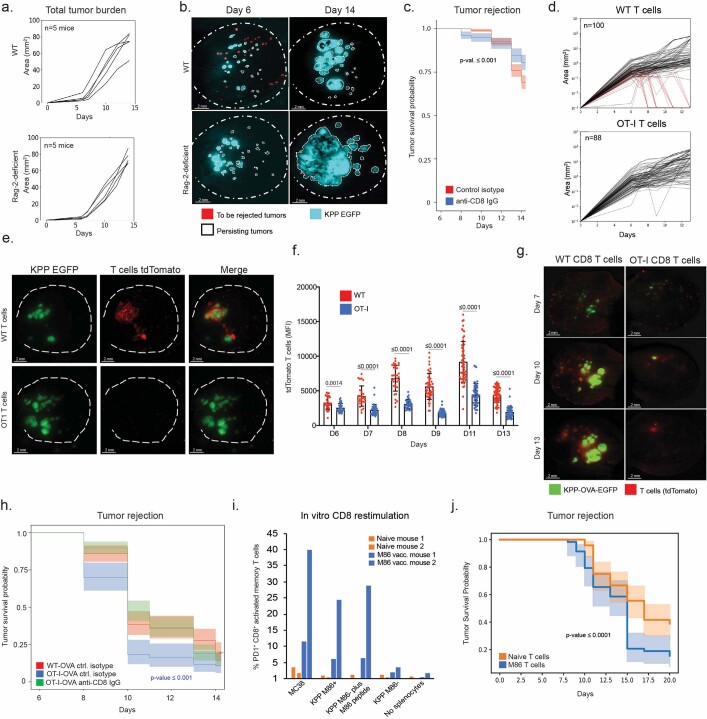
KPP STAMP microtumour rejection is mediated by tumour antigen specific CD8 T cells. **a**, Automated analysis of growth kinetics of KPP-EGFP total tumour array area (mm^2^) per animal in Rag-2-deficient and wild type animals as described in Fig. 1b, n = 5 animals per group. **b**, Representative images of KPP-EGFP tumour arrays in wild type (upper panel) or Rag-2-deficient (lower panel) mice at 6 and 14 days post tumour implantation related to **b**. Red encircled tumours are rejected and white encircled tumours are persistent between time points. **c**, Survival probability of individual tumours of KPP-EGFP arrays in mice treated with CD8 depleting (n = 318 tumours, 5 animals) or isotype control antibodies (n = 534 tumours, 5 animals). **d**, Automated analysis of growth kinetics of individual tumour area (mm^2^) for experiment as described in Fig. 1d. Red lines indicate tumours that are rejected, grey lines indicate tumours that persist. n = 100 tumours, n = 5 animals in Rag-2-deficient animals reconstituted with WT T cells, n = 88 tumours, n = 5 animals in Rag-2-deficient animals reconstituted with OT-I T cells. **e**, Representative images of STAMP tumour arrays for experiment as described in **d**. Red = T cells, green = KPP-EGFP. **f**, T cell infiltration kinetics of individual tumours measured by tdTomato MFI of T cells for experiment as described in **d**. n = 5 animals per group, n = 61 tumours in Rag-2-deficient animals reconstituted with WT T cells and n = 53 tumours in Rag-2-deficient animals reconstituted with OT-I T cells. Data are mean +/− s.e.m. Statistical analysis was performed using a two-tailed Mann-Whitney U-test. **g**, Representative image time course of KPP-OVA-EGFP tumour arrays in Rag-2-deficient animals reconstituted with wild type or OT-I T cells. n = 4 animals reconstituted with WT T cells, n = 5 animals reconstituted with OT-I T cells, n = 156 tumours in animals reconstituted with WT T cells, n = 93 tumours in animals reconstituted with OT-I T cells. Red = T cells, green=KPP-EGFP. **h**, Survival probability of individual tumours from mice bearing KPP-EGFP-OVA tumour in Rag-2-deficient animals reconstituted with wild type or OT-I T cells and treated with CD8 depleting or isotype control antibody. n = 4 WT-OVA isotype, n = 5 OT-I-OVA isotype and n = 5 OT-I-OVA anti-CD8 treated animals per group, n = 93 OT-I-OVA isotype, n = 156 WT-OVA isotype and n = 206 OT-I-OVA anti-CD8 treated tumours. Statistical analysis was performed with log-rank test (referenced to WT-OVA isotype). **i**, Frequency of tumour antigen (M86) specific T cells in M86-encoding RNA-LPX vaccinated or naive T cell donor mice shown as PD-1+ percent of CD8+ activated memory T cells. **j**, Survival probability of individual tumours from mice bearing KPP-M86-mTagBFP2 microtumours in Rag-2-deficient mice reconstituted with tumour antigen specific T cells (M86) from vaccinated mice or T cells from naive mice. n = 73 tumours in vaccinated animals, n = 72 tumours in naive animals, 4 animals per group pooled. **c,f,h,i**, *P* values are shown in the graph and colour-coded for the treatment group in **h**. **c,h,j**, The centre line shows the Kaplan-Meier curve, the shaded area shows the 95% confidence interval. Statistical analysis was performed with log-rank test. Source Data

To assess the role of adaptive immunity in controlling tumour growth, KPP-eGFP tumour arrays were implanted into wild-type or immunodeficient RAG2-deficient mice. Wild-type and RAG2-deficient mice displayed comparable initial tumour burdens (Extended Data Fig. 2a); however, after 14 days, immunocompetent mice demonstrated local rejection of around 30% of individual tumours from the same array, while immunodeficient mice did not reject the tumours (Fig. 1b,c and Extended Data Fig. 2b). Tumour survival analysis in wild-type and CD8-depleted mice confirmed that the anti-tumour immune response in STAMP involves CD8 T cells (Extended Data Fig. 2c). To assess the role of antigen-specific CD8 T cells in this local rejection, we implanted KPP-eGFP tumours in RAG2-deficient mice and reconstituted the mice with naive tdTomato^+^ T cells either from mice that contain a polyclonal population of T cells, or OT-I mice, which contain a monoclonal population of ovalbumin-specific CD8 T cells (Supplementary Video 1). In contrast to mice reconstituted with polyclonal T cells, antigen-mismatched T cells from OT-I mice did not mediate tumour rejection and were not substantially recruited to tumour sites (Fig. 1d and Extended Data Fig. 2d–f). As the KPP-eGFP tumour cells in this experiment do not express ovalbumin, we can conclude that bystander T cells were not sufficient to promote rejection of STAMP tumours.

These observations were corroborated by the converse experiment in which adoptive transfer of antigen-specific T cells was examined in mice that were implanted with KPP tumours expressing a model antigen (M86 tumour antigen or ovalbumin). As summarized in Extended Data Fig. 2g–j, reconstitution with antigen-specific CD8 T cells resulted in heightened T cell recruitment and local tumour rejection. Thus, our findings support a role for antigen-specific T cells recruited into STAMP tumours in mediating local rejection.

## Clinical relevance of STAMP phenotypes

**Fig. 2:**
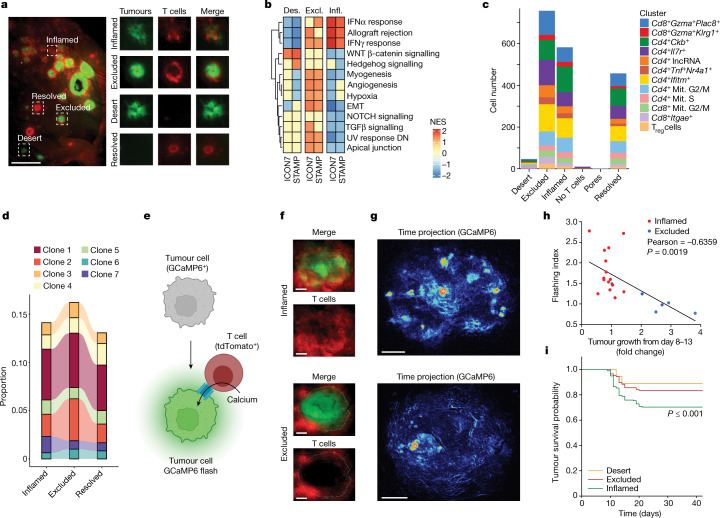
Immune-inflamed phenotype supports T cell effector function and tumour rejection. **a**, Representative image of a STAMP array of KPP-eGFP tumours at 8 days after tumour implantation in RAG2-deficient mice, reconstituted with tdTomato^+^ T cells. *n* = 50 mice, *n* = 10 independent experiments. Red, T cells; green, KPP-eGFP cells. Left, representation of the entire ear. Right, magnified images of individual tumours with different immune phenotypes. Scale bar, 2 mm. **b**, Heat map comparing the normalized enrichment scores for pathways that are significantly enriched across the immune-inflamed (infl.), immune-desert (des.) and immune-excluded (excl.) phenotypes from either human tumours from the ICON7 clinical trial or mouse STAMP tumours. Normalized enrichment scores were determined using clusterProfiler::GSEA using the false-discovery rate *P*-value adjustment method; *P*_ad__j_ < 0.2 was considered to be significant. **c**, The abundance of T cell subsets was determined using scRNA-seq analysis of STAMP tumour biopsies pooled by immune phenotype. T_reg_ cells, regulatory T cells. Mit., mitotic. **d**, The relative abundance of seven dominant T cell clonotypes across immune phenotypes. **e**, Schematic of cytotoxic T cell attack creating calcium-permeable pores in the tumour cell membrane, which triggers green fluorescence of the GCaMP6 calcium sensor in the tumour cell. **f**, Representative images of KPP-mTagBFP2-GCaMP6 STAMP tumours with the inflamed (top) or excluded (bottom) immune phenotype. *n* = 6 tumours. Red, T cells; green, GCaMP6. **g**, Time projection GCaMP6 fluorescent flashes of the tumour described in **f**. **h**, Correlation analysis of the GCaMP6 flashing index at 8 days after tumour implantation and the tumour growth fold change between day 8 and day 13 for immune-inflamed and immune-excluded tumours described in **f**. Pearson correlation was computed assuming a normal distribution. Statistical analysis was performed using two-tailed *t*-tests. **i**, Kaplan–Meier curve showing the survival probability of individual tumours of KPP-eGFP arrays that were immune phenotyped as immune-desert, immune-excluded or immune-inflamed by imaging 8 days after tumour implantation. *n* = 632 tumours, *n* = 10 mice. Statistical analysis was performed using a log-rank test (referenced to excluded tumours). For **f** and **g**, scale bars, 100 μm (inflamed) and 200 μm (excluded). Source Data

**Extended Data Fig. 3:**
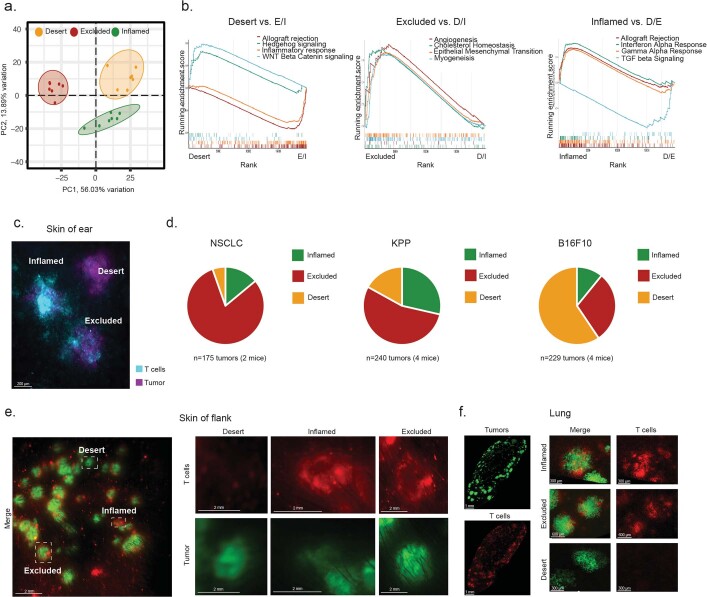
Transcriptionally distinct lesions with inflamed, excluded, and desert immune phenotypes coexist in tumour arrays. **a**, PCA of the top 1000 most variable genes of the bulk RNA-seq data of individually biopsied tumours with differential immune phenotype inflamed, desert, excluded. Ellipses at 95% confidence level are shown for each group. The percentage of explained variance for each principal component is annotated on the principal component axes. **b**, Detailed GSEA for selected pathways that are significantly enriched between three different immune phenotypes (inflamed, desert, excluded) from STAMP tumours. **c**, Representative STAMP tumour array of KPP-EGFP in Foxn1-Nude mice reconstituted with tdTomato +  T cells at 8 days post tumour implantation. Magenta = T cells, cyan = KPP-EGFP. n = 5 animals. **d**, Proportion of immune phenotypes present in STAMP arrays of three murine tumour models. NSCLC = non-small cell lung cancer (n = 175 tumours, n = 2 animals), KPP = pancreatic ductal adenocarcinoma (n = 240 tumours, n = 4 animals), and B16F10 = melanoma (n = 229 tumours, n = 4 animals). **e**, Representative images of STAMP tumour arrays of KPP-EGFP on abdominal skin of Rag-2-deficient mice reconstituted with tdTomato T cells, 8 days post tumour implantation, Red = T cells, green = KPP-EGFP. Overview of the entire abdominal tumour array (left panel), enlarged images of neighbouring tumours with inflamed, excluded, and desert immune phenotypes (right panels). n = 3 animals. **f**, Representative images of experimental lung metastases of KPP-EGFP in Rag-2-deficient mice reconstituted with tdTomato T cells, 8 days post tumour implantation. Red = T cells, green = KPP-EGFP. Overview of the entire lung lobe (left panel), enlarged images of metastatic lesions with inflamed, excluded, and desert immune phenotypes (right panel). n = 3 animals. Source Data

To further elucidate the drivers of local tumour rejection, we characterized the spatial distribution of T cells in individual tumours of the STAMP array using fluorescence microscopy. Despite being derived from a clonal tumour cell line, STAMP tumour array exhibited a combination of immune-inflamed, immune-excluded and immune-desert tumours at all of the analysed timepoints (Fig. 2a and Extended Data Fig. 3c). Moreover, we noted a fourth late-onset phenotype, termed resolved tumour, in which eGFP^+^ tumour cells disappeared leaving behind a cluster of tdTomato^+^ T cells.

The ability of STAMP to establish different tumour immune phenotypes (TIPs) provided the opportunity to characterize gene expression and pathways associated with each tumour niche. STAMP arrays were first imaged to classify tumours by immune phenotype and individual tumours were then isolated by punch biopsy for bulk RNA-sequencing (RNA-seq) analysis. Immune-desert, immune-excluded and immune-inflamed tumours were found to be transcriptionally distinct (Fig. 2b, Extended Data Fig. 3a and Supplementary Table 1). For each TIP, the patterns of gene expression in mouse STAMP tumours were notably similar to those observed in human tumours (ovarian, ICON7 phase III; bladder, IMvigor210 phase II) (Extended Data Fig. 3b, Supplementary Data 1 and Supplementary Table 2). Consistent with recent reports^11–15^, inflamed tumours were enriched for IFNα, IFNγ and allograft rejection signatures, and were also characterized by a downregulation of the TGFβ and NOTCH pathways^11,14^. Immune-excluded tumours were enriched for epithelial–mesenchymal transition (EMT), angiogenesis, hypoxia and ultraviolet light response signatures, suggesting enrichment in the stromal and myofibroblast components^12,13^. Finally, immune-desert tumours were enriched for WNT/β-catenin and Hedgehog pathways and showed a downregulation of immune signatures^14,15^. The similarity of gene signatures associated with the three TIPs across different human cancer types (ovarian, bladder and lung) and mouse STAMP tumours suggests that STAMP may recapitulate the general mechanisms underlying TIPs observed clinically.

To test whether the coexistence of different immune phenotypes is conserved across multiple tumour models and tissues in mice, we compared different tumour cell lines implanted with STAMP on the ear and on the flank, and in an experimental lung metastasis model (Extended Data Fig. 3c–f and Supplementary Video 2). We observed that, in each tumour model, neighbouring tumours could exhibit disparate phenotypes, including immune-inflamed, immune-excluded and immune-desert tumours. Notably, the relative frequencies of each phenotype varied depending on the cancer cell line used, confirming the importance of tumour cells themselves in influencing the probability of developing a certain TIP.

**Extended Data Fig. 4:**
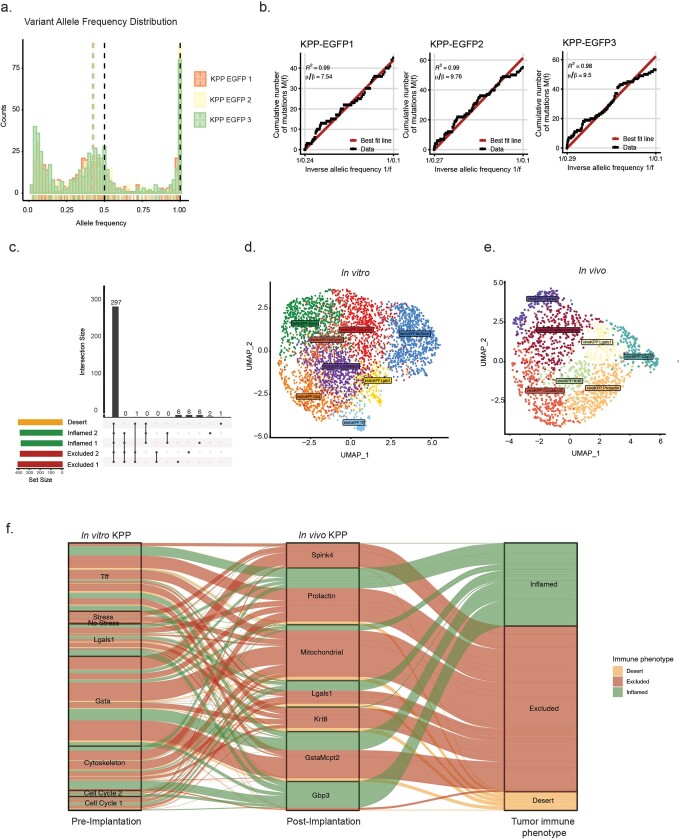
Inflamed, excluded, and desert immune phenotypes are not predetermined by genetic or non-genetic heterogeneity in the *in vitro* cultured cell line. **a**, Histogram depicts variant allele frequency (VAF) detected by whole exome sequencing of the KPP-EGFP cell line before implantation. n = 3 replicates indicated by colour with a corresponding dashed line showing the median VAF of the distribution. Homozygous and the heterozygous peaks indicated by black dashed lines. **b**, Neutrality test of low frequency variants for each replicate in **a**. The coefficient of determination (R^2^) is shown to indicate goodness of fit. The ratio of the mutation rate (μ) and the effective division rate (β) is the slope of the least squares fitted line according to the neutral cumulative mutation distribution. **c**, UpsetR plot showing the number of potentially explanatory variants detected by whole exome sequencing of pooled tumour biopsies with different immune phenotypes (x-axis). Variants have been confirmed after manual curation using detailed inspection of the alignment reads with Integrative Genome Viewer. **d**, UMAP embedding showing the KPP tumour cell clusters of scRNA sequencing data from *in vitro* cultured cells pre-implantation in STAMP. **e**, UMAP embedding showing the KPP tumour cell clusters of scRNA sequencing data from *in vivo* STAMP tumours post-implantation. **f**, Alluvial plot depicting the relationship between single cells from KPP-EGFP post implantation and its corresponding phenotypes together with the most likely *in vitro* cluster assignment as determined by SingleR.

Although the tumours in STAMP arrays shared the same clonal origin, it was possible that the different TIPs reflected rapid genetic divergence after implantation. To address this issue, we performed whole-exome sequencing (Extended Data Fig. 4a–c) and single-cell RNA-sequencing (scRNA-seq; Extended Data Fig. 4d–f) analysis of tumour cells before implantation and 3 weeks after implantation. As shown in Extended Data Fig. 4, neither pre-existing heterogeneity in the parental cell line nor genetic drift of the individual tumour cells growing in vivo correlated with the TIP diversity observed in STAMP. Thus, our findings indicate that clonally derived tumour cells have the unanticipated ability to develop heterogeneous immune phenotypes, demonstrating that the appearance of a given immune phenotype is not completely dependent on either host or tumour genetics.

## Inflamed niches support T cell function

**Extended Data Fig. 5:**
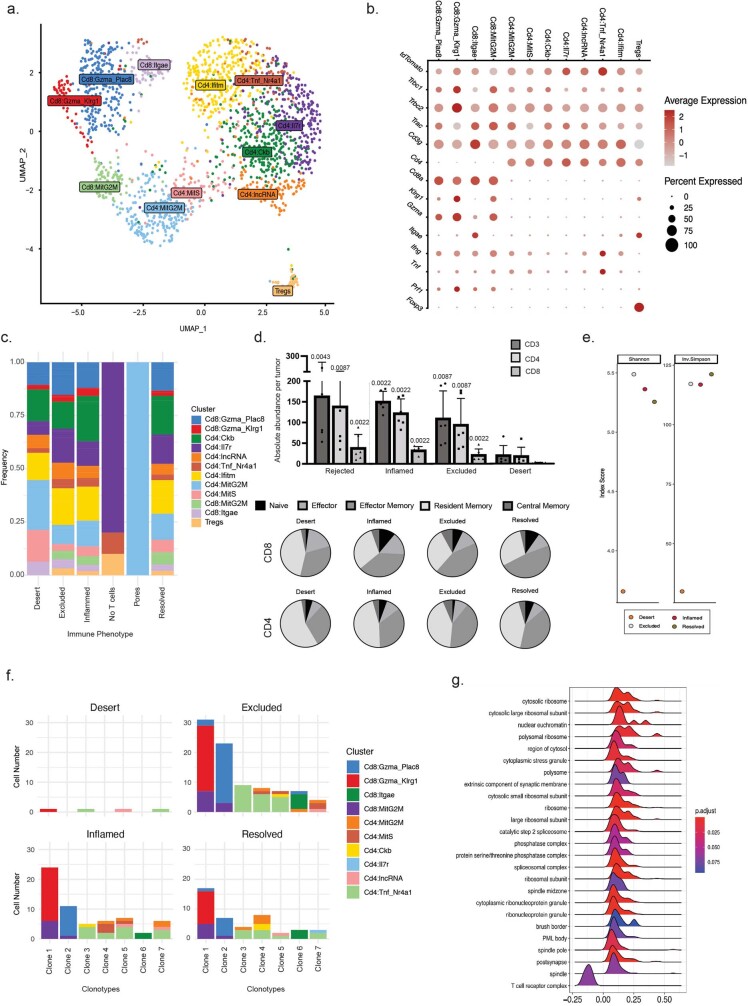
Desert, excluded or inflamed phenotypes are infiltrated by similar proportions of T cell subsets and clonotypes. **a**, UMAP embedding of tdTomato+ T cell subclusters (indicated by colours) from pooled STAMP tumour biopsies. **b**, Dot plot showing the relative expression of important marker genes within T cell subclusters. Relative expression level indicated by colour, and percent of cells expressing the transcript indicated by circle size. **c**, Relative abundance for each T cell subcluster separated by immune phenotype. **d**, Flow cytometry-based T-lymphoid immune cell profiling of rejected, inflamed, excluded and desert tumours at day 10. Absolute frequency of CD3, CD4 and CD8 T cells (upper plot) and proportion of naive, activated/resident, effector memory, and central memory of CD4 or CD8 T cells (lower plot). n = 6 animals with 3 tumours pooled per phenotype per animal. Data are mean +/−s.d. Statistical analysis was performed using a two-tailed t-test. *P* values are shown at the top of the graph. **e**, TCR clonotype diversity indices for each immune phenotype. **f**, Cell number of each T cell clonotype with cluster identification. **g**, Pathway analysis comparing excluded vs inflamed for combined top clonotypes. p-values were fdr adjusted and reported if p-value adjusted < 0.1. Source Data

We next examined whether the development of distinct immune phenotypes in a single tumour array reflected qualitative differences in T cell responses between neighbouring lesions. Although tumour-specific T cells in a single ear were probably derived from the same draining lymph nodes, differences in T cell infiltration and tumour growth control might have reflected differences in T cell receptor clonotype profiles. To this end, we performed 5′ scRNA-seq and T cell receptor sequencing analysis of individual tumours collected from the same mouse and compared T cells from immune desert, excluded, inflamed and rejected tumours (Fig. 2c,d). We identified 12 clusters of CD4 and CD8 T cells, including several clusters of effector T cells, T resident memory cells, regulatory T cells and mitotic cells (Fig. 2c, Extended Data Fig. 5a,b and Supplementary Table 3).

Notably, we did not observe any difference in the absolute or relative abundance of T cell subsets between TIPs on the basis of scRNA-seq or flow cytometry analysis (Fig. 2c and Extended Data Fig. 5c,d). Furthermore, we identified seven immunodominant clonotypes, the relative and absolute abundance of each was similar across TIPs (Fig. 2d and Extended Data Fig. 5e,f).

Although there were no differences in the overall abundance of T cell subsets or clonotypes, the transcriptional profiles of individual T cell clones between immune phenotypes exhibited differentially enriched pathways (Extended Data Fig. 5g, Supplementary Data 2 and Supplementary Table 4). Perhaps reflecting their participation in an ongoing immune response, T cell clones present in inflamed tumours were characterized by increased transcriptional and translational activity as well as mitochondrial biogenesis compared to the same T cells clones located in excluded tumours.

**Extended Data Fig. 6:**
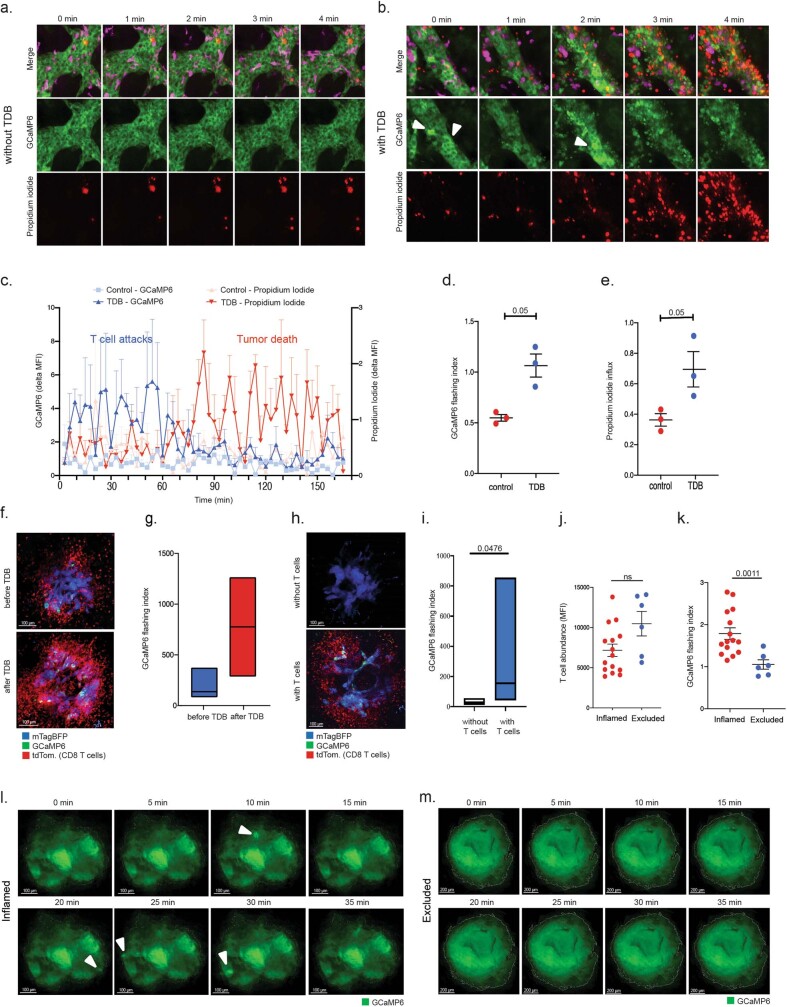
Spatial T cell patterns dictate efficiency of anti-tumour attack. **a-b**, Representative *in vitro* time-lapse image series of KPP-GCaMP6-Her2-expressing tumour organoids cultured with TdTomato T cells in the absence **(a)** or presence **(b)** of anti-Her2/anti-CD3 T cell-dependent bispecific antibody (TDB). Green = GCaMP6, red = propidium iodide, magenta = T cells. n = 3 biological replicates. Representative trace showing flashing behaviour (delta MFI) of GCaMP6 (blue) and influx of PI (red) fluorescence over time in the absence (control, light colour) or presence (TDB, dark colour) of TDB as described in **a** and **b. c**, Average GCaMP6 deltaMFI (blue) and average PI deltaMFI (red) plotted against the time (min) in the absence (control, light colour) or presence (TDB, dark colour) of TDB as described in **a** and **b**. Data are mean +/− s.e.m. **d**, GCaMP6 flashing index in the absence (control) or presence of TDB as described in **a** and **b**. Data are mean +/− s.e.m. Statistical analysis was performed using a one-tailed Mann-Whitney U-test. **e**, PI influx in the absence (control) or presence of TDB as described in **a** and **b**. Data are mean +/− s.e.m. Statistical analysis was performed using a one-tailed Mann-Whitney U-test. **f**, Representative *in vivo* 2-photon time-lapse images of KPP-GCaMP6-mTagBFP2-Her2 STAMP tumours in Rag-2-deficient mice 12 days after tumour cell seeding and adoptive transfer of tdTomato+ T cells. Imaging before intravenous (I.V.) administration of TDB (upper panel, n = 3 animals), and after I.V. administration of TDB (lower panel, n = 2 animals). Green = GCaMP6 (KPP), blue = mTagBFP2 (KPP), red = tdTomato (T cells). **g**. GCaMP6 flashing index of STAMP tumours before TDB or after TDB administration, as described in **f**. Green = GCaMP6 (KPP), blue = mTagBFP2 (KPP), red = tdTomato (T cells). The centre line shows the median, the box limits show the minimum and maximum values. **h**, Representative in vivo 2-photon time-lapse images of KPP-GCaMP6-mtagBFP2-Her2 STAMP tumours in Rag-2-deficient mice with (upper panel) or without (lower panel) reconstitution with tdTomato + T cells and 12 days after tumour cell seeding. Green = GCaMP6 (KPP), blue = mTagBFP2 (KPP), red = tdTomato (T cells). n ≧ 3 animals. **i**, GCaMP6 flashing index of STAMP tumours in Rag-2-deficient mice with and without adoptive transfer of tdTomato+ T cells, as described in **h**. The centre line shows the median, the box limits show the minimum and maximum values. Statistical analysis was performed using a two-tailed Mann-Whitney U-test. **j**, T cell abundance (MFI) of inflamed and excluded KPP-GCaMP6-mtagBFP2-Her2 STAMP tumours in Rag-2-deficient mice 8 days after tumour cell seeding and adoptive transfer of tdTomato+ T cells. n = 6 excluded and n = 15 inflamed tumours. Data are mean +/− s.e.m. Statistical analysis was performed using a two-tailed Mann-Whitney U-test. **k**, GCaMP6 flashing index of inflamed and excluded STAMP tumours, as described in **j**. Data are mean +/− s.e.m. Statistical analysis was performed using a two-tailed Mann-Whitney U-test. **l**, Representative in vivo epifluorescence time-lapse images of GCaMP6-expression of inflamed KPP tumours. n = 15 tumours. Green = GCaMP6. **m**, Representative *in vivo* epifluorescence time-lapse images of GCaMP6-expression of excluded KPP tumours. n = 6 tumours. Green = GCaMP6. **d, e, i, j, k**, P values are shown at the top of the graph. Source Data

As mitochondrial dynamics influence T cell fate^16–18^ and translational activity correlates with the activation and differentiation states of effector T cells^19^, we investigated the functionality of the T cells present in immune-inflamed versus immune-excluded tumours. We engineered a clonal KPP tumour cell line expressing GCaMP6 calcium sensor^20^ that emits green fluorescence after effector T cell attack (Fig. 2e). We validated our experimental approach by demonstrating that calcium flashing occurs as a result of a T cell attack in organoids in vitro (Extended Data Fig. 6a–e and Supplementary Videos 3 and 4) and in vivo (Extended Data Fig. 6f,g and Supplementary Videos 5–7). In a RAG2-deficient model with and without T cell reconstitution, we show that T cells are required for calcium flashing in tumour cells (Extended Data Fig. 6h,i).

We next quantified differences in calcium flashing between STAMP tumours within a single array. Higher flashing indices were found in inflamed tumours (Fig. 2f–h, Extended Data Fig. 6j–m and Supplementary Videos 8 and 9) and corresponded to a slow tumour growth rate (Fig. 2h). Thus, inflamed tumours that nurture effector T cell function were more likely to regress compared with immune-excluded or immune-desert tumours (Fig. 2i).

Taken together, these results indicate that T cells of the same TCR clonotype exhibit an improved functional capacity when localized in immune-inflamed tumours, emphasizing a determinative role for the tumour microenvironment in shaping the activity and fate of endogenous effector T cells.

## Myeloid–stroma control of immune phenotype

**Fig. 3:**
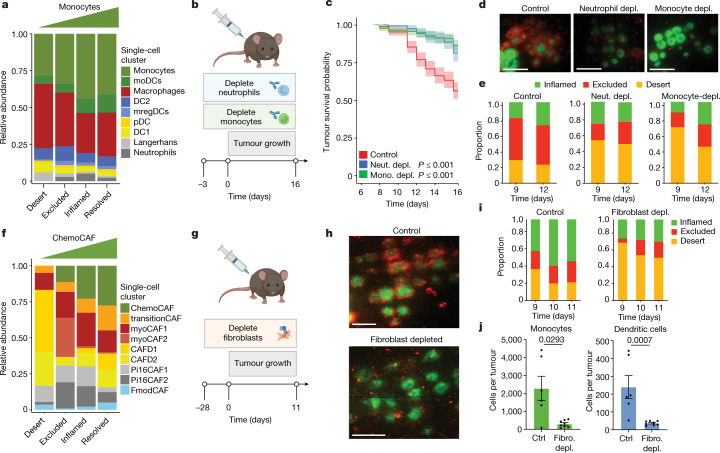
Myeloid and stromal cells control TIP and tumour fate. **a**, The relative abundance of myeloid cell subclusters as determined by scRNA-seq analysis of STAMP tumour biopsies pooled by immune phenotype. DCs, dendritic cells; moDCs, monocyte-derived dendritic cells; mregDCs, mature DCs with immunoregulatory molecules; pDCs, plasmacytoid dendritic cells. **b**, The experimental design relating to **c**–**e**. Neutrophils or monocytes were ablated using depleting antibodies (Gr1 or Ly6C, respectively) beginning 3 days before STAMP implantation of KPP-eGFP tumour arrays in E8I CD8-cre LSL-tdTomato immunocompetent mice. *n* = 6 isotype-control-treated mice, *n* = 319 tumours; *n* = 5 neutrophil-depleted (neut. depl.) mice, *n* = 187 tumours; *n* = 5 monocyte-depleted (mono. depl.) mice, *n* = 301 tumours. **c**, The survival probability of individual tumours of KPP-eGFP arrays related to **b**. The centre line shows the Kaplan–Meier curve and the shaded area shows the 95% confidence interval. Statistical analysis was performed using log-rank tests (referenced to the isotype control). **d**, Representative image of STAMP tumour arrays of non-depleted (left) neutrophil-depleted (middle) or monocyte-depleted (right) mice related to **b** at 11 days after tumour implantation. Red, T cells; green, KPP-eGFP. Scale bars, 2 mm. **e**, The proportion of immune-inflamed, immune-excluded and immune-desert tumours related to **b**–**d**. **f**, The relative abundance of fibroblast subclusters determined by scRNA-seq analysis of STAMP tumour biopsies pooled by immune phenotype. **g**, The experimental design relating to **h**–**j**. DPT^+^ fibroblasts  were ablated by tamoxifen and diphtheria toxin administration in Dpt-cre-ERT2 LSL-DTR mice before STAMP implantation of KPP-eGFP tumour arrays into mice reconstituted with tdTomato^+^ T cells. *n* = 5 control mice, *n* = 207 tumours; *n* = 7 fibroblast-depleted mice, *n* = 314 tumours. **h**, Representative image of STAMP tumour arrays of control (top) or fibroblast-depleted (bottom) mice at 11 days after tumour implantation. Red, T cells; green, KPP-eGFP. Scale bars, 1 mm. **i**, The proportion of Immune-inflamed, immune-excluded and immune-desert tumours related to **g**. **j**, Flow-cytometry-based monocyte and dendritic cell profiling of tumours in fibroblast-depleted versus control non-depleted mice related to **g**. Data are mean ± s.e.m. Statistical analysis was performed using a two-tailed Mann–Whitney *U*-test. Source Data

**Extended Data Fig. 7:**
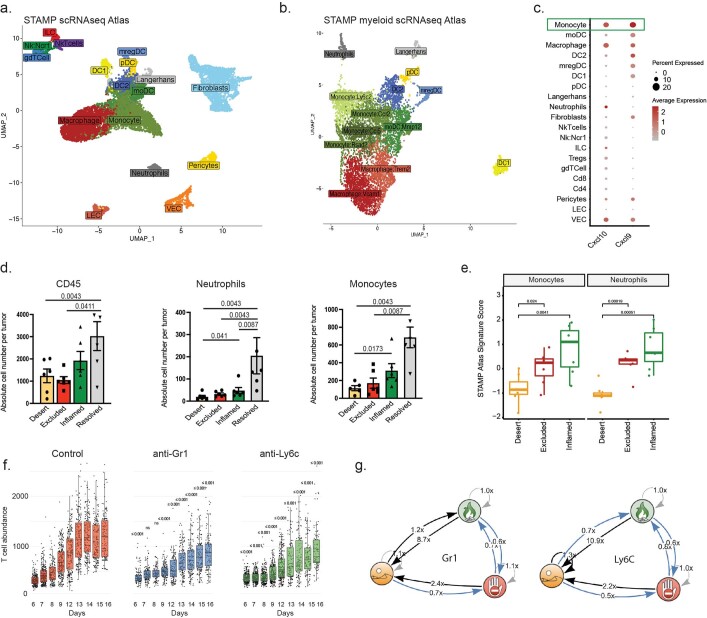
Myeloid depletion reduces T cell recruitment and transition towards inflamed phenotype. **a**, STAMP scRNA-seq atlas UMAP embedding of innate, myeloid, and stromal cell clusters from STAMP single tumour biopsies pooled per immune phenotype. **b**, UMAP embedding for the myeloid compartment of the scRNA from single tumour biopsies pooled per immune phenotypes. **c**, Dot plot for expression of Cxcl9 and Cxcl10 across all cell clusters of the scRNA sequencing STAMP tumour atlas described in **a**. **d**, Flow cytometry-based myeloid cell profiling of 5 pooled tumour biopsies per immune phenotype at 10 days post tumour implantation. n = 6 animals. Data are mean +/− s.e.m. Statistical analysis was performed using a two-tailed Mann-Whitney U-test. *P* values are shown at the top of the graphs. **e**, Scores of myeloid signatures in bulk RNA-seq of tumours at 8 days post-implantation. n = 7 tumours per group (gene signatures for each myeloid population have been derived from the scRNA-seq myeloid STAMP Atlas). Box plots show quartiles of the dataset and whiskers show the rest of the distribution. Statistical analysis was performed using a two-tailed t-test with unequal variances, adjusted for false discovery rate. P values are at the top of the graphs. **f**. Total T cell abundance in individual tumours over time in control, Ly6C- and Gr1-depleted animals as described in Fig. 3b, n = 6 Isotype control-treated animals, n = 5 Ly6c- and n = 5 Gr1-depleted animals, n = 319 Isotype control-treated, n = 301 Ly6c- and n = 187 Gr1-depleted tumours. Box plots show quartiles of the dataset and whiskers show the rest of the distribution, excluding points determined to be outliers by exceeding 1.5x the interquartile range. Statistical analysis was performed using a two-tailed t-test (referenced to Isotype control treated tumours). P values are shown in the graph. **g**, Analysis of immune phenotype transition dynamics of tumours in Isotype control-treated, Ly6C- and Gr1-depleted animals related to **f**. Arrows represent fold changes in transition likelihood between control and depleted animals. Black = increased transition, blue = decreased transition. Source Data

**Extended Data Fig. 8:**
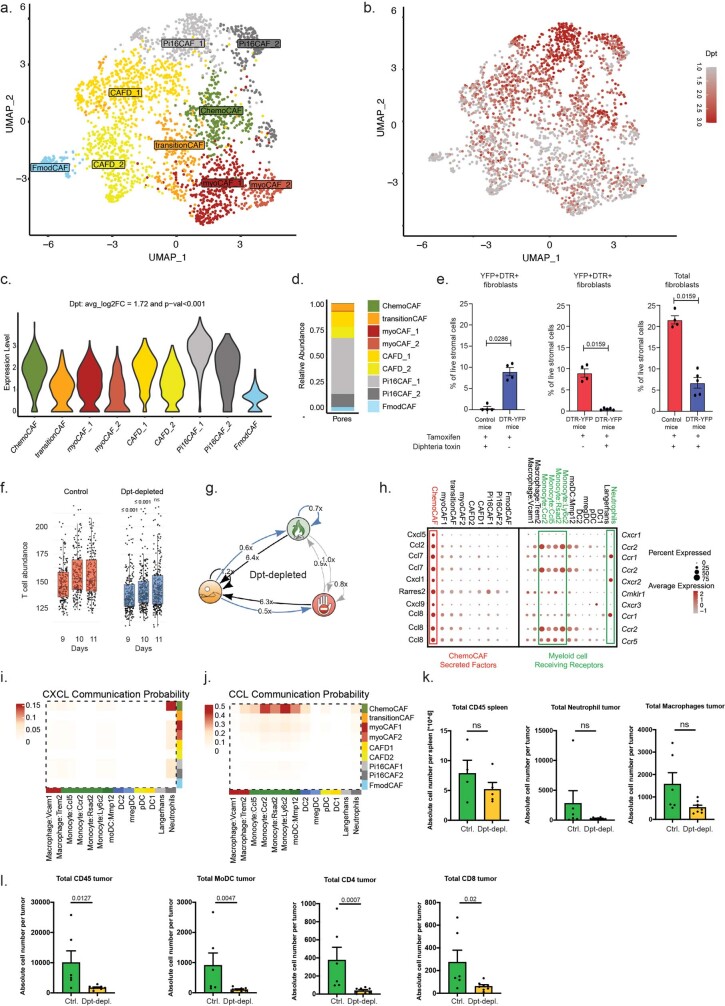
Depletion of Dpt+ fibroblasts promotes immune desert phenotype in STAMP. **a**, UMAP embedding for the fibroblast compartment for the scRNA from pooled single tumour STAMP biopsies. **b**, Dermatopontin (Dpt) expression overlaid on the fibroblast UMAP. **c**, Violinplot showing the expression levels of Dpt across fibroblast subclusters. **d**, Barplot showing the relative abundance of the fibroblast subclusters for microporated skin. **e**, Quantification of fibroblast frequency in skin of Dpt-CreERT2_LSL-DTR-YFP mice with and without Tamoxifen and/or Diphtheria toxin (DTX) treatment. n = 4 Tamoxifen/DTX-treated Control mice, n = 4 Tamoxifen-treated DTR-YFP mice and n = 5 Tamoxifen/DTX-treated DTR-YFP mice. Data are mean +/− s.e.m. Statistical analysis was performed using a two-tailed Mann-Whitney U-test. **f**, Total T cell abundance in individual tumours over time in control and Dpt-depleted animals as described in Fig. 3h. n = 5 control and n = 7 Dpt-depleted animals, n = 207 control and n = 314 Dpt-depleted tumours. Box plots show quartiles of the dataset and whiskers show the rest of the distribution, excluding points determined to be outliers by exceeding 1.5x the interquartile range. Statistical analysis was performed using a two-tailed t-test. **g**, Analysis of immune phenotype transition dynamics of tumours in Dpt-depleted animals related to **f**. Arrows represent fold changes in transition likelihood between control and depleted animals. Black = increased transition, Blue = decreased transition. **h**, Dot plot for expression of ligand-receptor pairs of the main secreted factors of the ChemoCAF for all of the fibroblast and myeloid populations from the scRNA sequencing STAMP tumour atlas. **i-j**, Communication probability estimated by CellChat for **i**, CXCL chemokine pathway with fibroblasts subclusters as the sender populations and myeloid subclusters as the receiver and **j**, CCL chemokine pathway with fibroblasts subclusters as the sender populations and myeloid subclusters as the receiver. **k-l**, Flow cytometry-based immune cell profiling of tumours in Dpt-depleted versus non-depleted animals. n = 6 control animals and n = 8 DTR animals. Data are mean +/− s.e.m. Statistical analysis was performed using a two-tailed Mann-Whitney U-test. **k**, Shows cell types with no significant change upon depletion. **l**, Shows cell types with significant differences between control and depletion. **e,f,k,l**, *P* values are shown at the top of the graphs. Source Data

To examine the role of the tumour microenvironment in the development of TIPs, we created a scRNA-seq atlas of STAMP microtumours at early timepoints after T cell infiltration (Extended Data Fig. 7a), focusing on myeloid (Extended Data Fig. 7b and Supplementary Table 5) and stromal cell subsets (Extended Data Fig. 8a and Supplementary Table 6). We found that monocytes and monocyte-derived cells were primary producers of the T cell chemoattractants CXCL9 and CXCL10 (Extended Data Fig. 7c) and that increased monocyte and neutrophil abundances were associated with the inflamed and resolved TIPs (Fig. 3a); similar results were obtained both by flow cytometry and deconvolution of bulk RNA-seq (Extended Data Fig. 7d,e). We demonstrated a functional role for these myeloid cell subsets in the recruitment and spatial patterning of T cells by depleting Ly6C^+^ monocytes  or Ly6G^+^ neutrophils (Fig. 3b and Supplementary Data 3). Notably, depletion of either myeloid population resulted in a significant decrease in tumour rejection (Fig. 3c), which was accompanied by a strong decrease in overall T cell recruitment and an increase in desert tumours (Fig. 3d,e and Extended Data Fig. 7f,g).

Further examination of the STAMP atlas revealed an enrichment of a unique subset of cancer-associated fibroblasts (CAFs) that we named ChemoCAFs in the inflamed and resolved tumours (Fig. 3f and Extended Data Fig. 8a). ChemoCAFs abundantly express chemokines, but lack expression of inflammatory cytokines, such as IL-1, IL-6, IL-11, LIF, CSF-2 or VEGFa, which are classically produced by inflammatory CAFs. ChemoCAFs also express dermatopontin (DPT), which is a known marker of PI16^+^ fibroblasts, the primary fibroblast population at steady state in the skin^21^ (Extended Data Fig. 8b–d) and the progenitors of CAF subsets in tumours^21^. We therefore used an inducible mouse model of DPT^+^ fibroblast depletion^21^ to eliminate around 75% of the skin fibroblasts at the time of tumour implantation to inhibit the emergence of CAFs after STAMP tumour implantation (Fig. 3g, Extended Data Fig. 8e and Supplementary Data 4) and follow subsequent immune cell recruitment. In the absence of fibroblasts, STAMP microtumours exhibited a strong decrease in overall T cell recruitment and an increase in immune-desert tumours (Fig. 3h,i and Extended Data Fig. 8f,g), emphasizing a role for the fibroblasts of the tumour microenvironment in shaping the immunological infiltrate of STAMP tumours.

Given that fibroblast and monocyte/neutrophil depletion both produced an enrichment in the immune-desert phenotype, we hypothesized that there might be a mechanistic relationship between the two cell types. Importantly, 8 out of the 10 top marker genes defining the ChemoCAF population are chemoattractants for monocytes and neutrophils (CCL7, CCL2, CXCL1, CXCL5, CCL8, chemerin and CXCL12; Extended Data Fig. 8h). We used a cell–cell communication database, CellChat^22^, which predicted a role for ChemoCAFs in recruiting monocytes and neutrophils (Extended Data Fig. 8i,j). Using flow cytometry, we confirmed experimentally that there was deficient myeloid and T cell recruitment in the CAF-depleted tumour microenvironment but not in a secondary lymphoid organ such as the spleen (Fig. 3j and Extended Data Fig. 8k,l).

Together, these results suggest that the early fibroblast niche signals to myeloid cells (including neutrophils and monocytes) that in turn promote T cell recruitment and infiltration. T cell recruitment into an inflamed tumour microenvironment supports their effector function and increases the probability that a given microtumour will be rejected.

## Early inflammation predicts rejection

**Extended Data Fig. 9:**
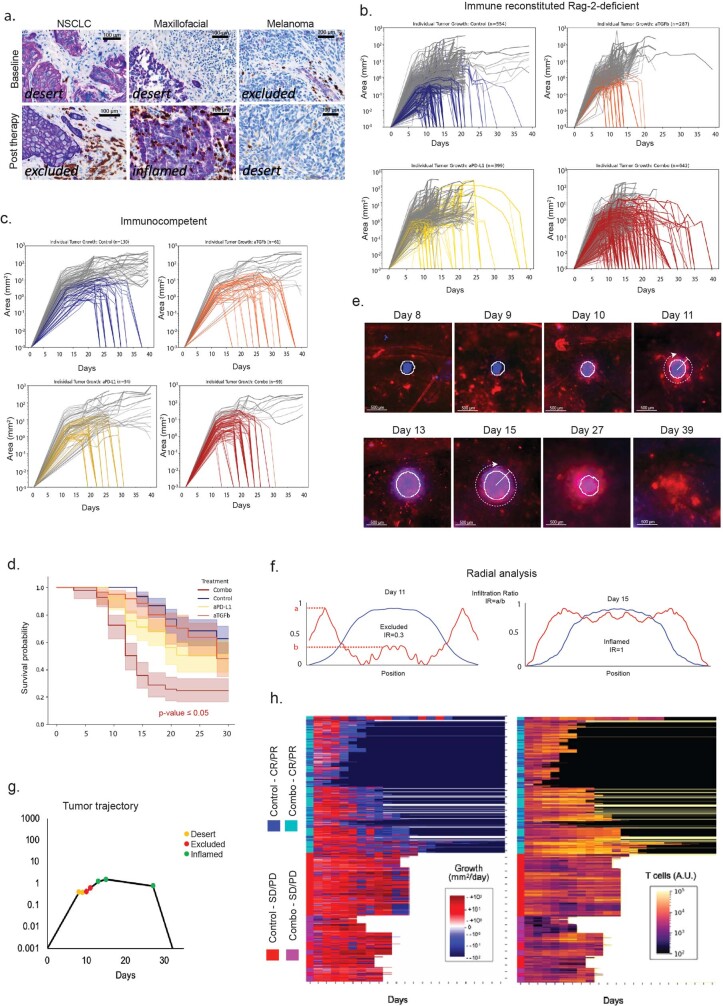
Early transition to an inflamed phenotype predicts tumour response to immunotherapy. **a**, Histology of patient paired tumour biopsies at baseline and at progression after treatment with checkpoint blockade. CD8 staining in brown, pan-cytokeratin staining in magenta. **b**, Individual tumour growth kinetics of KPP-GFP STAMP tumours for experiment with immune reconstituted animals described in Fig. 4a. **c-d**, STAMP arrays of KPP-EGFP were implanted in immunocompetent animals and treated 10 days post-implantation with isotype control antibodies (n = 130 tumours, 4 animals), anti–PD-L1 (n = 94 tumours, 4 animals), anti–TGF-β (n = 61 tumours, 3 animals) or a combination of anti–PD-L1 with anti–TGF-β (n = 99 tumours, 4 animals). **c**, Individual tumour growth kinetics (mm^2^) shown with coloured lines to indicate tumours that are rejected and grey lines to indicate tumours that persist. **d**, Survival probability of individual tumours described for **c** and **d**. The centre line shows the Kaplan-Meier curve, the shaded area shows the 95% confidence interval. Statistical analysis was performed with log-rank test. *P* value is shown in the graph and colour-coded for the treatment group. **e**, Image series of individual STAMP tumour over time. Red = T cells, blue = tumour cells. **f**, Quantification of median radial fluorescence profile for individual tumour shown in **e** to distinguish non-desert, T cell excluded phenotype (low T cell infiltration ratio, day 11) and T cell inflamed phenotype (high T cell infiltration ratio, day 15). **g**, Overlay of the automated classification of immune phenotypes with the individual tumour growth curve for the example in **e**. **h**, Hierarchical clustering of individual tumour immune growth rates (left) and T cell infiltration (right) for isotype control treated tumours and combo anti–PD-L1/anti–TGF-β treated tumours as described in Fig. 4a. Black = tumour resolved, white = mouse death/euthanasia. Cyan=combination treated responders (C.R. + P.R.), magenta = combination treated non-responders (S.D. + P.D.), blue = Control responders (C.R.+ P.R.), red = control non-responders (S.D. + P.D.). Source Data

Finally, we investigated how the spatial distribution of T cells predicts tumour progression or rejection during immunotherapy. Although tumours exhibit one or another immune phenotype, it is unclear whether these states are stable or dynamic over time. In humans, repeat biopsies before and after treatment can reveal alterations in immune phenotype (Extended Data Fig. 9a and Supplementary Table 7), but it is difficult to know whether these changes reflect an overall alteration in TIPs as opposed to pre-existing spatial heterogeneity^23,24^. By contrast, STAMP provides a unique opportunity to determine how immune phenotypes evolve spontaneously or after therapy.

**Fig. 4:**
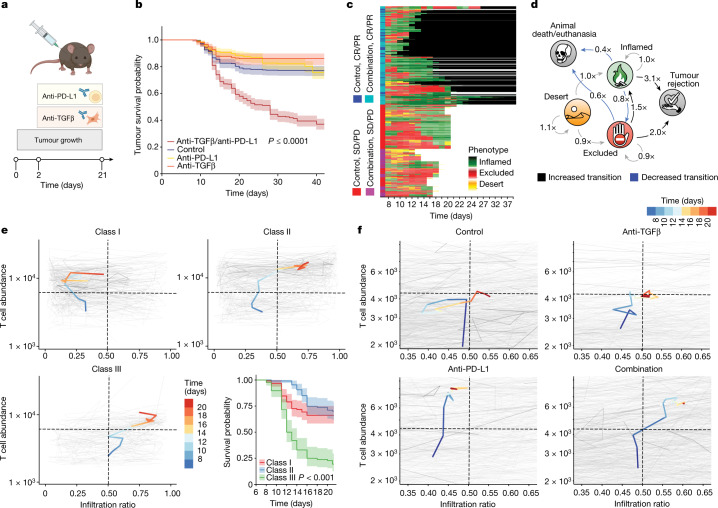
Early transition to an immune-inflamed phenotype predicts tumour response to immunotherapy. **a**, The experimental design relating to the experiments shown in **b**–**f**. KPP-eGFP STAMP tumour arrays were implanted into RAG2-deficient mice reconstituted with tdTomato^+^ T cells and treated at day 2 after implantation with isotype control antibodies (*n* = 554 tumours, *n* = 9 mice), anti-TGFβ (*n* = 287 tumours, *n* = 5 mice), anti-PD-L1 (*n* = 399 tumours, *n* = 6 mice), or a combination of anti-PD-L1 and anti-TGFβ (*n* = 642 tumours, *n* = 11 mice). **b**, The survival probability of individual tumours of KPP-eGFP arrays related to **a**. Statistical analysis was performed using a log-rank test (referenced to the isotype control). **c**, Hierarchical clustering of individual tumour trajectories related to **a**, showing immune phenotypes over time for tumours treated with isotype control or a combination of anti-PD-L1 and anti-TGFβ antibodies. Black, tumour resolved; white, mouse death/euthanasia; cyan, combination-treated responders (complete responders and partial responders (CR/PR)); magenta, combination-treated non-responders (stable disease and progressive disease (SD/PD)); blue, control responders (CR/PR); red, control non-responders (SD/PD). **d**, Markov chain showing the fold difference in the probabilities of transition between TIPs for combination anti-PD-L1/anti-TGFβ treatment versus the control condition. Bold indicates increased transition. Blue indicates decreased transition. ‘*×*’ is the fold change. **e**, Unsupervised clustering of individual tumour immune trajectories highlighting changes in T cell abundance and infiltration ratio over time. *n* = 6 isotype-treated control mice. *n* = 321 tumours. The median immune trajectory for each of the three identified classes is shown in bold, and the colour scale indicates time. The survival probability of individual tumours grouped by immune-trajectory class is shown at the bottom right. Statistical analysis was performed using a log-rank test (referenced to class I tumours). **f**, Immune trajectories of individual tumours grouped by treatment related to **a**. Median immune trajectories are shown in bold, and the colour scale indicates time. In **b** and the bottom right of **e**, the centre line shows the Kaplan–Meier curve and the shaded area shows the 95% confidence interval. Source Data

As immune-excluded and immune-desert STAMP tumours exhibited an upregulation in the TGFβ pathway (Fig. 2b) and a combination of TGFβ and PD-L1 inhibition has previously shown anti-tumour efficacy in mice^11,25^, we examined the effects of these agents in STAMP (Fig. 4a). In contrast to single-agent treatment, the combination therapy of anti-TGFβ and anti-PD-L1 antibodies led to an improved overall response rate in KPP-eGFP STAMP tumours (Fig. 4b and Extended Data Fig. 9b–d).

**Extended Data Fig. 10:**
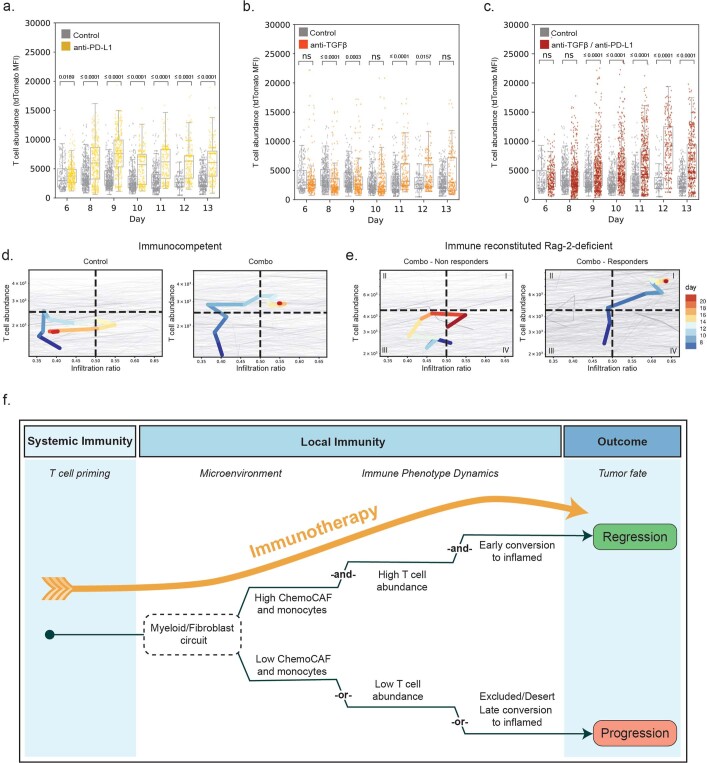
Early transition to an inflamed phenotype predicts tumour response to immunotherapy. **a-c**, Total T cell abundance (tdTomato MFI) for individual microtumours as described in Fig. 4a. Isotype control antibodies (n = 554 tumours, 9 animals, grey dots), **a**, anti–PD-L1 (n = 399 tumours, 6 animals, yellow dots), **b**, anti– TGF-β (n = 287 tumours, 5 animals, orange dots), or a **c**, combination of anti–PD-L1 with anti–TGF-β (n = 642 tumours, 11 animals, red dots). Box plots show quartiles of the dataset and whiskers show the rest of the distribution, excluding points determined to be outliers by exceeding 1.5x the interquartile range. Statistical analysis was performed using a two-tailed Mann-Whitney U-test. P values are shown in the graphs. **d**, Immune-histories of individual KPP-GFP STAMP tumours (grey lines) implanted in E8I.CD8A.Cre-Rosa26.LSL.tdTomato animals and treated at day 10 post-implantation with isotype control antibodies or combo anti-PD-L1/anti-TGF-b. n n ≧ 86 tumours per group and 3-4 animals per group. Average tumour trajectory changing T cell abundance (y-axis) and inflammation ratio (x-axis) over time for each treatment condition is shown in bold, with colour scale indicating time. **e**, Immune-histories for individual tumours of combination treated animals grouped as responders or non-responders for experiment described in Fig. 4a. Average immune-history for each class is shown in bold, with colour scale indicating time. **f**, Graphical summary of immune dynamics leading to tumour rejection or progression. Source Data

To better understand how T cell dynamics are associated with tumour rejection, we examined the spatial distribution of tdTomato^+^ T cells over the lifetime of around 2,000 tumours using a high-content image analysis pipeline (Extended Data Fig. 9e–g). The immune history of individual tumours was plotted using a hierarchically clustered heat map to create representations of TIP, tumour growth rate and total T cell abundance over time in control and combination treated mice (Fig. 4c and Extended Data Figs. 9h and 10a–c). We found that TIPs are dynamic and can interconvert over time both in control and treated mice.

To quantify the effect of the combination therapy on the spatial distribution of T cells, we modelled phenotype transitions as a Markov chain to measure the probability of transition between the different states: immune-desert, immune-excluded, immune-inflamed, rejected tumour and mice death/euthanasia. We then compared the Markov chain for combination-treated mice with the control (Fig. 4d, Supplementary Table 8 and Supplementary Data 5). We observed that the combination treatment increased the probability of transition from immune-excluded to immune-inflamed tumours and decreased the probability of reverse transition from immune-inflamed to immune-excluded tumours. Moreover, the probability of an immune-inflamed or an immune-excluded tumour becoming resolved was increased relative to control if treated with combination immunotherapy. These results highlight the overall dynamics of transitions between immune states promoted by combination immunotherapy.

To complement the Markov analysis and link the immunomodulatory effect of the treatment with its efficacy, we evaluated whether the history of an individual tumour might predict its eventual likelihood of rejection. We first defined the infiltration ratio for each tumour over time by measuring the T cell abundance in the tumour core relative to the total T cell abundance (Extended Data Fig. 9f). We then plotted the total T cell abundance against the T cell infiltration ratio for each individual tumour at every timepoint and performed unbiased clustering of the individual tumour trajectories. Unbiased clustering revealed three classes of tumour trajectories in the control condition (class I, II and III; Fig. 4e). Class I defines tumours that increase T cell abundance but progress as immune-excluded tumours; class II defines tumours transitioning first through an immune-excluded state before ultimately reaching a late inflamed phenotype; and class III defines tumours moving early to an immune-inflamed phenotype with a high T cell abundance and infiltration ratio. Notably, class III was the only trajectory associated with a strong enrichment in spontaneous tumour rejection, suggesting that considering the history of a TIP is essential to predict outcome.

To determine the treatment effect, we grouped individual tumour trajectories according to treatment, and represented the average trajectory for each treatment group (Fig. 4f and Extended Data Fig. 10d). Combination-treated tumours displayed a steady, strong increase in the total T cell abundance, and an inflamed T cell infiltration ratio similar to the class III trajectory. Tumours treated with anti-PD-L1 therapy were also marked by a strong increase in T cell abundance; however, they kept a very low infiltration ratio and persisted mostly as immune-excluded tumours similar to the class I trajectory. By contrast, anti-TGFβ-treated tumours displayed the lowest T cell recruitment and infiltration ratio, similar to the control group.

Finally, we evaluated the trajectories of responders versus non-responder tumours in the combination therapy and showed that responding tumours followed the class III trajectory (Extended Data Fig. 10e) associated with an early infiltration of T cells in the tumour core, whereas the non-responders persisted with a low T cell abundance and low infiltration ratio. These results illustrate how combination immunotherapy pushes tumours towards the path of early inflammation leading to rejection, and they highlight the predictive value of TIP history.

## Conclusion

Here we describe a preclinical model to characterize the spatiotemporal patterns of an antitumour immune response. STAMP revealed that genetically identical clonal tumours generate dynamic CD8 T cell infiltrations that are heterogenous between adjacent tumours. Longitudinal monitoring of the local immune response revealed that tumour rejection is associated with the early transition towards an immune-inflamed phenotype, characterized by heightened T cell cytotoxicity and a local decrease in TGFβ. We show that T cell infiltration is controlled in time and space by the tumour microenvironment (a summary is provided in Extended Data Fig. 10f) such as chemokine-producing CAFs and myeloid cells, both important players to recruit and support T cell function locally in inflamed tumours leading to tumour rejection. This study supports the role of an early inflamed TIP as a predictive biomarker of response to immunotherapy in patients by nurturing the function of newly expanded T cells. However, this immune phenotype is not stable over time, highlighting the importance of tracking the immune history of tumours in patients. Understanding the spontaneous or cancer immunotherapy-induced evolution of TIPs in the clinic will be an important consideration in predicting the likelihood of clinical response and providing biomarkers that may guide therapy in patients with cancer.

## Methods

### STAMP implantation

Experimental procedures were performed in 8–12-week-old male mice under anaesthesia (60–100 mg per kg ketamine and 5–10 mg per kg xylazine, intraperitoneal (i.p.) injection). Before microporation, the ear hair was removed using Veet depilatory cream, then rinsed with water, dried and the dorsal side of the ear was immobilized with double-sided tape to expose the ventral side. Microporation at 71 µm depth and 9% pore density was performed by applying the P.L.E.A.S.E. laser device (Pantec Biosolutions) to the ventral side of the ear using a custom program previously validated for the cell line. After the microporation process, 150 µl of a tumour cell suspension (EMT6-eGFP, CT26-RFP, 4T1-mTagBFP2, KPP-eGFP, KPP-mTagBFP2, KPP-eGFP-OVA, NSCLC-EGFP or B16F10) at 40–80 × 10^6^ cells per ml diluted in PBS was applied to the ear covering the ventral side. Cells were incubated for 30 min then the excess cell suspension was removed, pores were covered with Matrigel (Corning) and incubated for 15 min until Matrigel polymerized. Microtumour growth becomes evident 5 to 12 days after tumour implantation, depending on the cell line. Once tumours were visibly observed, the mice were observed and tumours were measured at least twice per week. Monitoring frequency was increased if any additional adverse effects were observed, up to daily depending on the severity, according to the direction of the veterinary staff. The mice were euthanized immediately if the tumour area reached or exceeded 70% or the total ear surface, or if tumours fell outside the IACUC Guidelines for Tumours in Rodents. Moreover, if the ears were in a condition that compromises normal ear and bodily function as determined by the veterinary staff, the mice were euthanized. Any mice exhibiting other unexpected adverse effects such as severe hunching or severe lethargy or any moribund mice were euthanized immediately. Mice were weighed at least weekly and those mice losing >15% body weight were weighed daily. Animals that had lost 20% body weight were euthanized or brought to the attention of the veterinary staff. Mice with a body condition score of 2 or less out of 5 were euthanized.

### Cell lines

B16F10, EMT6, CT26 and 4T1 mouse parental lines were sourced from the ATCC. KPP (PDAC) and NSCLC cells were derived from primary tumours of Genentech Cancer Immunology GEMM mice. Parental cell lines were further engineered in house using PiggyBac vectors to overexpress different types of fluorescent reporters (eGFP, RFP or mTagBFP2) or/and model antigens (ovalbumin or decamer peptide of tumour neoantigens, including the M86 neoantigen). Genentech built a centralized cell bank, gCELL, to support the needs of cell-based research within Genentech. gCELL is tasked to bank verified, quality-assured cell lines for distribution throughout Genentech. This provides a consistent source of cell lines for all levels of research to enable experimental reproducibility and access to baseline information such as morphology, growth conditions, RNA-seq and whole-exome sequencing derived from these lines. gCELL also provides an important mechanism to ensure that cell lines are used in accordance with all terms and conditions. All stocks are tested for mycoplasma before and after cells are cryopreserved. Two methods were used to avoid false-positive/negative results: Lonza Mycoalert and Stratagene Mycosensor. All of the cell lines tested negative for mycoplasma.

### Mice

Mice were housed under specific-pathogen-free conditions at the Genentech animal facility. Mice were maintained in accordance with the Guide for the Care and Use of Laboratory Animals (National Research Council, 2011). Genentech is an AAALAC-accredited facility and all animal activities in this research study were conducted under protocols approved by the Genentech Institutional Animal Care and Use Committee (IACUC). Mice were housed in individually ventilated cages within animal rooms maintained under a 14 h–10 h light–dark cycle. Animal rooms were temperature and humidity controlled between 68 and 79 °F (20.0 to 26.1 °C) and from 30% to 70%, respectively, with 10 to 15 room air exchanges per hour. Male mice (aged 8–12 weeks) that appeared healthy and free of obvious abnormalities were used for the study. B6.Cg-Foxn1nu/J (000819), C57BL/6-Tg (CAG-EGFP)1Osb/J (003291) and C57BL/6J (000664) and B6(Cg)-Ifnar1 tm1.2Ees/J (028288) mice were purchased from Jackson Laboratories. B6.129S6-Rag2^tm1Fwa^ N12 (RAGN12), C.Cg/AnNTac-Foxn1^nu^ NE9 (BALBNU-M) and BALB/cAnNTac (BALB-M) mice were purchased from Taconic Biosciences.

CD4.cre.tg Rosa26.LSL.tdTomato.cki OT-I.TCR.tg (*OT-I*^*−/−*^ and *OT-I*^*+/+*^), DPT-IRES-Cre.ERT2. ki.B6N.1C9-1-H4-1_Rosa26.LSL.YFP.cki.B6J_Rosa26.LSL.DTR.cki and E8I.CD8A.IRES.GFP.Cre.tg Rosa26.LSL.tdTomato.cki mice were bred in house.

### Subcutaneous tumours

For subcutaneous tumour inoculation, mice were injected subcutaneously with 0.5 × 10^6^ KPP-eGFP cells in 100 μl of a 1:1 dilution of PBS and Matrigel (Corning). Once the tumours were visibly observed, the mice were observed and tumours were measured at least twice per week. The monitoring frequency was increased if any additional adverse effects were observed, up to daily depending on severity, according to the directions of the veterinary staff. Mice were euthanized immediately if the tumour volume exceeds 2,000 mm^3^, or if tumours fell outside the IACUC Guidelines for Tumours in Rodents. Any mice exhibiting other unexpected adverse effects such as severe hunching or severe lethargy or any moribund mice were euthanized immediately. Mice were weighed at least weekly and those mice losing >15% body weight were weighed daily. Mice that lost 20% body weight were euthanized or brought to the attention of the veterinary staff. Mice with a body condition score of 2 or less out of 5 were euthanized.

### In vivo cell depletion

#### Myeloid depletion

E8I.CD8A.IRES.GFP.Cre.tg Rosa26.LSL.tdTomato.cki mice were treated i.p. with 300 µg per mouse of anti-mouse Ly6C/G, anti-mouse Ly6C antibodies or isotypes controls from BioXcell (BE0075 and BE0203, respectively) for 3 consecutive days before KPP-eGFP STAMP tumour implantation. To maintain the cell depletion, the antibody treatment was sustained until the end of the study. Depletion was validated using flow cytometry. Tumour growth and T cell infiltration was monitored daily by GFP/tdTomato imaging.

#### CD8 and CD4 T cell depletion

Mice were treated with 100 µg per mouse i.p. of anti-mouse CD8b (BE0223) and anti-mouse CD4 (BP-0003-3) or isotype control: (1) C57BL/6J mice were CD8 and CD4 depleted for 3 consecutive days before KPP-eGFP STAMP tumour implantation. Tumour growth was followed daily by direct imaging. (2) DPT.IRES.Cre.ERT2.ki. B6N.1C9-1-H4-1_ Rosa26.LSL.YFP.cki. B6J_Rosa26.LSL.DTR.cki mice were injected with anti-CD8 and anti-CD4 antibodies for 3 consecutive days. After 14 days (when antibody was cleared) the DTP-diphtheria-toxin-induced depletion experiment started. All CD4 and CD8 depletions were confirmed using flow cytometry.

#### Fibroblast depletion

As a DPT expression control, DPT.IRES.Cre.ERT2.ki. B6N.1C9-1-H4-1_Rosa26.LSL.YFP.cki. B6J_Rosa26.LSL.DTR.cki mice, previously CD4/CD8 depleted, were injected daily with 20 µg per mouse i.p. of tamoxifen. After DPT–YFP expression was established by flow cytometry, mice were treated with diphtheria toxin every other day starting at 4 days before KPP-eGFP STAMP tumour implantation and tdTomato^+^ T cell reconstitution. Depletion was validated using flow cytometry. Tumour growth and T cell infiltration was monitored daily by GFP/tdTomato imaging.

#### Adoptive transfer of antigen-specific T cells

B6.129S6-Rag2tm1Fwa N12 mice were adoptively transferred with 4 × 10^6^ *OT-I*^*−/−*^ tdTomato^+^ T cells or 4 × 10^6^ *OT-I*^*+/+*^ tdTomato^+^ T cells after KPP-EGFP-OVA expressing cells were implanted. Tumour growth and T cell infiltration was monitored daily by GFP/tdTomato imaging

### Cancer immunotherapy treatment

#### Antibody treatment

Mice were implanted with STAMP microtumours as described above. Mice were distributed into treatment groups to exclude cage effects and, when possible, to account for differential initial tumour growth. Treatment was initiated 1 day after tumour implantation for immunodeficient mice or 7 days after tumour implantation for immunocompetent mice. Mice were treated every other day with isotype control antibodies (anti-gp120; mouse IgG, 3E5, 10 mg per kg), anti–PD-L1 (mouse IgG1, 6E11, 10 mg per kg first dose followed by 5 mg per kg thereafter), anti-TGFβ (mouse IgG1, 1D11, 10 mg per kg) or a combination of anti-PD-L1 with anti-TGFβ. Beginning 5 days after treatment initiation, mice were imaged daily using the M205FA stereoscope with a ×1.0 PlanApo lens (10450028; Leica Microsystems) and the ORCAII Digital CCD (Hamamatsu Photonics) to monitor tumour growth and T cell progression. For selected experiments, immunodeficient mice were injected intravenously with 4 × 10^6^ isolated tdTomato^+^ T cells from CD4.cre.tg_Rosa26.LSL.tdTomato.cki_OT-I.TCR.tg mice as described in the figure legends. Individual microtumours were biopsied individually or pooled by phenotypes.

#### Tumour antigen vaccination

The KPP-GFP clonal cell line was CRISPR-edited to ablate GFP expression, and then transfected with a PiggyBac vector to express a decamer peptide of tumour neoantigens, including the M86 neoantigen, and cytoplasmic mTagBFP2 (EF1A_MC38-Deca16-cBFP). M86 RNA-lipoplex (RNA-LPX) vaccines were assembled from M86-coding RNAs synthesized by Genentech with liposomes consisting of DOTMA and DOPE at a charge ratio (+):(−) of 1.3:2.0 as described previously^26^. CD4.cre.tg Rosa26.LSL.tdTomato.cki T cell donor mice (*n* = 2) were intravenously vaccinated with M86 RNA-lipoplex 1, 2 and 3 weeks before T cell isolation using the EasySep Mouse T Cell Isolation Kit (StemCell Technologies) from the spleen. T cells were used for adoptive transfer into RAG2-deficient mice or in vitro stimulation assays. To validate RNA-LPX-induced antigen-specific T cells, 200,000 splenocytes from vaccinated (*n* = 2) or naive (*n* = 2) mice were co-cultured with 40,000 tumour cells expressing (+) or not (−) the tumour neoantigens. As a positive control, M86 peptide was added to the negative tumour cells. Antigen-specific T cell stimulation was measured by flow cytometry as PD-1^+^ percentage of live, CD45^+^CD90.2^+^CD8^+^CD44^+^SlamF7^+^ cells. STAMP tumour assays were performed as described in RAG2-deficient mice with 4 × 10^6^ tdTomato^+^ CD3^+^ T cells isolated as described below from vaccinated or naive mice.

### Metastasis mouse model and imaging of whole-mount tissue

RAG2-deficient mice were injected intravenously with 0.1 × 10^6^ KPP-eGFP cells and 4 × 10^6^ tdTomato^+^ CD3^+^ T cells isolated as described below. Then, 8 days after intravenous injection, the mice were euthanized, and 20 ml of cold PBS/heparin 5 IU ml^−1^ solution was perfused directly into the right ventricle using a 27-gauge needle. Lungs were isolated by dissection and tissues were fixed using 4% paraformaldehyde in PBS^23^. Tissue clearing was performed using the FluoClearBABB approach^27^ and whole-mount images were then acquired using a SP8 microscope equipped with a white-light laser and the HCX APO L ×20/0.95 NA IMM lens (Leica Microsystems). Imaging data were analysed on a workstation (Thinkmate) using Imaris software (Bitplane).

### T cell isolation

T cells were isolated from C57BL/6J or CD4.cre.tg Rosa26.LSL.tdTomato.cki OT-I.TCR.tg (*OT-I*^*−/−*^ and *OT-I*^*+/+*^) mice. Spleens were collected and dissociated with the end of a plunger from a 1 ml syringe into 10 ml of PBS before filtration through a 70 μm cell strainer. T cells were isolated for intravenous injection using the EasySep Mouse T Cell Isolation Kit (StemCell Technologies). A total of 4 × 10^6^ isolated T cells was injected intravenously per mouse.

To generate cytotoxic T cells from OT-I mice, splenocytes were isolated and stimulated with 10 nM OVA257-264 peptide (AnaSpec) in complete medium containing Gibco RPMI 1640 (Thermo Fisher Scientific) with 10% Gibco fetal calf serum (Thermo Fisher Scientific), 2 mM l-glutamine, 50 IU ml^−1^ Gibco penicillin–streptomycin (Thermo Fisher Scientific) and 50 μM β-mercaptoethanol. After 3 days of stimulation, cells were resuspended in complete medium with 10 IU ml^−1^ recombinant human IL-2 (rHIL-2). Cytotoxic T cells were kept at a density of 0.5 × 10^6^ cells per ml and fresh complete medium with rHIL-2 was added every 48 h. Cytotoxic T cells were used between 6 and 8 days after primary in vitro stimulation.

### Flow cytometry

Ear tissue was isolated using a 1 mm Miltex sterile disposable biopsy punch with plunger (Integra Biosciences) from mice bearing STAMP microtumours or mock-implanted control mice at either 8 or 18 days after tumour implantation. Tumours were digested in 500 µl (STAMP microtumours) or 3,000 ml (subcutaneous) of PBS containing 0.1 mg ml^−1^ DNase I (Roche) and collagenase D at 1 mg ml^−1^ (Roche) for 30 min at 37 °C to obtain a single-cell suspension.

For surface staining, cells from digested tumours were incubated with Fc block (5 µg ml^−1^, BD Biosciences, 2.4G2) and stained with antibody mix for 30 min and Viability Dye eFluor 780 (eBioscience).

Antibodies were used at 1:200 dilution. Anti-mouse CD19 (B4), anti-mouse I-A/I-E (M5/114.15.2), anti-mouse F4/80 (BM8), anti-mouse/human CD11b (M1/70), anti-mouse Ly-6G (1A8), anti-mouse Ly-6C (HK1.4), anti-mouse CD69 (H1.2F3), anti-mouse CD25 (PC61), anti-mouse CD4 (RM4-5), anti-mouse CD62L (MEL-14), and anti-mouse/human CD44 (IM7) antibodies were purchased from BioLegend. Anti-mouse CD45 (30:F11), anti-mouse CD86 (GL1), CD11c anti-mouse (N418), and anti-mouse CD8a (53-6.7) antibodies were purchased from Thermo Fisher Scientific. Anti-mouse CD3 (17A2) antibodies were purchased from BD Biosciences.

Live CD45^+^ singlet cell subsets were gated as follow: MHC class II^+^CD11c^+^F4/80^−^ (dendritic cells) or CD103^+^ or CD86^+^ (activated dendritic cells); CD11c^−^CD11b^+^F4/80^+^ (macrophages); CD11b^+^Ly6G^+^Ly6C^int^ (neutrophils); CD11b^+^Ly6G^low^Ly6C^+^ (monocytes); CD3^+^ T cells were divided as CD3^+^CD4^+^ T cells, CD3^+^CD8^+^ T cells, CD3^+^CD69^+^ activated/resident T cells, CD3^+^CD44^+^CD62L^−^ effector/effector memory T cells or CD3^+^CD44^−^CD62L^+^ naive T cells.

Flow cytometry data were collected using the BD LSRFortessa cell analyser (BD Biosciences) and analysed using FlowJo Software (v.10.2; FlowJo).

### STAMP microtumour sample preparation for bulk RNA-seq of tumour biopsies

FOXN1-deficient nude mice with adoptively transferred tdTomato^+^CD3^+^ T cells bearing KPP-eGFP STAMP microtumours were biopsied 8 days after tumour implantation using a 1 mm Miltex sterile disposable biopsy punch with plunger (Integra Biosciences). Each tissue biopsy was transferred into a separate 1.5 ml tube (Eppendorf) containing 0.25 ml Invitrogen TRIzol Reagent (Thermo Fisher Scientific). Biopsies were incubated in TRIzol for 5 min with intermittent vortexing. A total of 50 µl chloroform (MilliporeSigma) was added to the homogenate, vortexed for 20 s and incubated at 20–25 °C for 2–3 min. To accelerate phase separation, the samples were centrifuged at 10,000*g* for 18 min at 4 °C. The aqueous (top) phase was removed by aspiration and transferred to a clean 1.5 ml tube (Eppendorf). A volume of 100% RNase-free ethanol (MilliporeSigma) equal to the volume of the aqueous layer was added, and the RNA was further isolated using the RNeasy Micro Kit (Qiagen). Alternatively, an individual tumour biopsy was immersed in RNA later and the RNA was further extracted using the RNeasy Mini Kit (Qiagen).

### Sample preparation of sorted STAMP microtumours for scRNA-seq

FOXN1-deficient nude mice with adoptively transferred tdTomato^+^ CD3^+^ T cells bearing KPP-eGFP STAMP microtumours were biopsied 8 days after tumour implantation using a 1 mm Miltex sterile disposable biopsy punch with plunger (Integra Biosciences). Then, 3–6 pooled tissue biopsies were moved into a precooled 1.5 ml tube (Eppendorf) containing 300 µl digestion cocktail consisting of Gibco RPMI 1640 (Thermo Fisher Scientific), 0.1% Gibco fetal calf serum (Thermo Fisher Scientific), 0.1 mg ml^−1^ Liberase (Roche), 0.1 mg ml^−1^ DNase I (Roche) and 32 µM Gibco actinomycin D (Thermo Fisher Scientific). Tissues were incubated for 30 min at 37 °C and 950 rpm on a Thermoblock (Eppendorf) and mechanically dissociated every 10 min with a pipette. To quench the digestion, the cell suspension was filtered through a 40 µm mesh into a precooled fluorescence-activated cell sorting (FACS) filter tube containing quenching buffer of Gibco fetal calf serum (Thermo Fisher Scientific) with 32 µM Gibco actinomycin D (Thermo Fisher Scientific). The cell suspension was centrifuged at 350*g* for 8 min and resuspended in 400 µl Gibco RPMI 1640 (Thermo Fisher Scientific) with 5 µM Calcein Blue (Invitrogen) and a 1:200 dilution of the Molecular Probes Fixable Live/Dead Near-IR Dead Cell Stain Kit (Thermo Fisher Scientific). FACS analysis was performed to isolate Calcein-Blue-positive and L/D NearIR-negative cells into a precooled 1.5 ml Eppendorf tube containing 750 µl collection buffer consisting of Gibco RPMI 1640 (Thermo Fisher Scientific), 10% Gibco fetal calf serum (Thermo Fisher Scientific), 32 µM Gibco actinomycin D (Thermo Fisher Scientific). The cell number and viability were determined on the Vi-Cell XR cell viability analyser (Beckman Coulter) and scRNA-seq library preparation was performed using the dual single-cell mouse kit 5′/TCR according to the manufacturer’s instructions (10x Genomics).

### 3D in vitro tumouroid cultures/co-cultures

KPP mouse pancreatic cancer cells expressing human HER2 and cytosolic GCaMP6 were suspended in three-dimensional collagen matrices as described previously^28^. In brief, a solution of rat-tail collagen I (MilliporeSigma) was brought to a neutral pH on ice and mixed with KPP.hHer2.GCaMP6 cells to a final concentration of 2 mg ml^−1^ collagen and 1.0 × 10^4^ cells. Then, 150 µl of this suspension was added to individual wells of an eight-chambered cover glass (Cellvis). The chambers were incubated at 20–25 °C for 15 min then incubated at 37 °C with 5% CO_2_ for an additional 15 min. After incubation, 500 µl of complete medium containing Gibco RPMI 1640 (Thermo Fisher Scientific) with 10% Gibco fetal calf serum (Thermo Fisher Scientific), 2 mM l-glutamine and 50 IU ml^−1^ Gibco penicillin–streptomycin (Thermo Fisher Scientific) was carefully added to each well. Cells were allowed to grow in collagen matrices for 5 days before imaging.

3D imaging of OT-I T cells interacting with KPP.hHer2.GCaMP6 tumouroids in collagen matrices was performed on the TiE microscope (Nikon) with the CSU-X1 Spinning Disk (Yokogawa Electric) and Prime sCMOS camera (Photometrics). The medium in the eight-chamber imaging slides containing tumour cell collagen matrices was replaced with Gibco RPMI 1640 with no phenol red (Thermo Fisher Scientific) with 10% Gibco fetal calf serum (Thermo Fisher Scientific), 2 mM l-glutamine and 50 IU ml^−1^ Gibco penicillin–streptomycin (Thermo Fisher Scientific) with 3 μM propidium iodide (Thermo Fisher Scientific). OT-I cells were prelabelled with Celltrace FarRed (Thermo Fisher Scientific) according to the manufacturer’s protocol. FarRed-labelled OT-I cells were added to each chamber containing collagen-suspended KPP.hHer2.GCaMP6 tumouroids and allowed to infiltrate for 2 h before imaging. At the time of imaging, T-cell-dependent bispecific antibody (anti-hHER2::anti-CD3e) was added to the chambers at a final concentration of 500 nM to induce cytotoxic T cell recognition of hHER2-expressing cancer cells. In the control conditions, no antibody was added.

### STAMP microtumour correlative imaging of Ca^2+^ influx and T cell infiltration

RAG2-deficient mice with adoptively transferred tdTomato^+^ CD3^+^ T cells bearing mTagBFP2 and GCaMP6-expressing KPP STAMP microtumours were anaesthetized by isoflurane inhalation to effect and imaged daily from 4 days after tumour implantation to 15 days after tumour implantation at the same ear regions. Epifluorescence time-lapse microscopy image series were acquired daily at the same ear regions with the ×1.0 Leica PlanApo objective (Leica 10450028) on the Leica M205 FA epifluorescence stereomicroscope every minute for 60–70 min. Image analysis was performed using Imaris (Bitplane). Time-lapse image series of individual tumours at 8 days after tumour implantation were semi-automatically segmented and analysed for Ca^2+^ influx between timepoints. Furthermore, tumour sizes, T cell abundances and T cell infiltrations were analysed. Time-lapse image sequences of individual tumours at 13 days after tumour implantation were semi-automatically segmented and were analysed for tumour size to determine the tumour growth from day 8 to day 13.

### STAMP microtumour correlative imaging of Ca^2+^ influx and T cell infiltration after TDB administration

FOXN1-deficient nude mice with adoptively transferred tdTomato^+^CD3^+^ T cells bearing mTagBFP2 and GCaMP6-expressing KPP STAMP microtumours were anaesthetized by isoflurane inhalation to effect and imaged 12 days after tumour implantation. Image series were acquired every 90 s for 45 min using a two-photon laser-scanning microscope (Ultima In Vivo Multiphoton Microscopy System, Bruker Technologies) with alternating excitation from dual Ti:sapphire lasers (MaiTai DeepSee, Spectra Physics) tuned to 830 nm and 980 nm, and a ×16/0.8 NA immersion objective lens (Nikon). Thereafter, T-cell-dependent bispecific antibodies (anti-hHER2::anti-CD3e) were administered intravenously (6 mg per kg) and multiphoton time-lapse microscopy image acquisition was continued at the same region. Time-lapse image series of individual tumours were semi-automatically segmented with Imaris (Bitplane) and analysed for Ca^2+^ influx between timepoints.

### Immunophenotypes in the imCORE Paired Biopsy trial

Tumour biopsies were obtained from patients enrolled in the imCORE^29^ Paired Biopsy trial (NCT03333655) between January 2018 and March 2020. This study is an ongoing, open-label, multicentre trial initiated in February 2018 and conducted globally including study centres in the USA, France and Spain. Adult patients with metastatic cancer or haematological malignancies who demonstrated clinical benefit on cancer immunotherapy and had a tumour biopsy both at baseline (pre-treatment/archival) and at progression were eligible for inclusion. Cancer immunotherapy included marketed agents (including those targeting CTLA-4, PD-L1 or PD-1) or those administered through participation in a Roche/Genentech CPI clinical trial. Patients with the best overall response (on the basis of Response Evaluation Criteria in Solid Tumours v.1.1) of complete response, partial response or stable disease after >6 months (or >3 months if enrolled under an earlier protocol version) were eligible. PanCK/CD8 dual staining was performed on histological sections from baseline and progression formalin-fixed paraffin-embedded tumour samples. Immune phenotypes were determined by a pathologist (Histogenex) using defined criteria^30^.

### Clinical trials

Clinical trials registrations were as follows: IMvigor210 (NCT02951767/NCT02108652), ICON7 (NCT00483782) and imCORE trial (NCT03333655). For the ICON7 and IMvigor210 studies, the full protocols are available at https://clinicaltrials.gov/. For the imCORE trial, the protocol is available on request (www.roche.com/about_roche/roche_worldwide.htm, +1 888-662-6728, global-roche-genentech-trials@gene.com). For the IMvigor210 trial, the study was approved by the independent review board at each participating site and was performed in full conformance of the provisions of the Declaration of Helsinki and Good Clinical Practice Guidelines. Approval from the Institutional Ethics Committee or the Institutional Review Board was obtained before the study start and was documented in a letter to the investigator specifying the date on which the committee met and granted the approval. The ICON7 protocol was compliant with good clinical practice guidelines and the Declaration of Helsinki. Approval by ethics committees was obtained at each clinical site, nationally or both. For the imCORE trial, the study protocol was approved at enrolling institutions and by local ethics committees (Sarah Cannon Research Institute, WIRB; IUCT Oncopole Toulouse, France; Clinica Universidad di Navarra, Spain).

The ICON7 and IMvigor210 trials have been previously published^11,13^. For the imCORE trial, the patients were recruited by participating institutions if eligibility criteria (including clinical benefit from checkpoint inhibition and biopsies were available before and after treatment from the same tissue) were met. No knowledge of immunophenotype was known at the time of recruitment, therefore limiting potential bias. Clinical characteristics of imCORE patients are summarized in the Supplementary Table 7.

All patients have provided written informed consent.

### Image analysis

#### Ca^2+^ influx index in vivo epifluorescence microscopy

An isosurface is created that matches the tumour-associated mTagBFP2 fluorescence of individual STAMP microtumours. The sum of mTagBFP2 and GCaMP6 fluorescence pixel intensities is calculated for each channel for the tumour isosurface for each timepoint. The absolute delta of the sum fluorescence intensities between consecutive timepoints is calculated, averaged for each fluorophore and normalized to the respective mean fluorescence intensity (MFI). The Ca^2+^ influx index is the result of dividing the normalized average delta sum of GCaMP6 intensities by the normalized average delta sum of mTagBFP2 intensities.

#### T cell abundance

An isosurface is created that matches the tumour-associated mTagBFP2 fluorescence of individual STAMP microtumours. The MFIs for the T cell-associated tdTomato fluorescence are determined for the tumour isosurface for each timepoint. The T cell abundance index is the result of calculating the median of the tdTomato MFIs across all timepoints.

#### T cell infiltration index

An isosurface is created that matches the tumour-associated mTagBFP2 fluorescence of individual STAMP microtumours. Using the tumour isosurface, two new regions are defined: the tumour centre (central 50% of tumour isosurface) and the tumour periphery (the area surrounding the tumour that is up to 50 µm distance from tumour border). The MFIs for the T-cell-associated tdTomato fluorescence are determined for the tumour centre and the tumour periphery for each timepoint, and the median of MFIs across all timepoints is calculated. The T cell infiltration index is the result of the ratio of those medians (centre/periphery).

#### Ca^2+^ influx index in vivo two-photon microscopy

An isosurface is created that matches the tumour-associated mTagBFP2 fluorescence of individual STAMP microtumours. The sum of mTagBFP2 and GCaMP6 fluorescence pixel intensities are calculated for the tumour isosurface for each timepoint. The absolute delta of the sum fluorescence intensities between consecutive timepoints is calculated, averaged for each fluorophore and normalized to the respective mean MFI across all timepoints. The normalized average delta sum of GCaMp6 intensities is divided by the normalized average delta sum of mTagBFP2 intensities (value1). Also, the average s.d. of mTagBFP2 and GCaMP6 fluorescence of every pixel of the tumour isosurface is calculated across the time series. The average s.d. of GCaMP6 is divided by the average s.d. of mTagBFP2 (value2). The Ca^2+^ influx index (two-photon) is the result of multiplying value1 and value2.

#### Ca^2+^ influx index in vitro spinning-disk confocal microscopy

An isosurface is created that matches the tumour-associated GCaMP6 background fluorescence of tumour cells. The median of GCaMP6 MFIs is calculated for the tumour cell isosurfaces for each timepoint. The absolute delta of the median MFIs between consecutive timepoints is calculated and averaged. The Ca^2+^ influx index (spinning-disk confocal microscopy) is the result of dividing the average delta median GCaMP6 MFIs by the mean GCaMP6 MFI across all timepoints.

#### PI influx index in vitro spinning disk confocal microscopy

An isosurface is created that matches the tumour-associated GCaMP6 background fluorescence of tumour cells. The median of propidium iodide MFIs is calculated for the tumour cell isosurfaces for each timepoint. The absolute delta of the median MFIs between consecutive timepoints is calculated and averaged. The propidium iodide influx index is the result of dividing the average delta median propidium iodide MFIs by the mean propidium iodide MFI across all timepoints.

#### U-net model training

Images of tumour fluorescence were binned and resized to 512 × 512 px using custom FIJI scripts. Binary (two-class) masks were manually generated with 1 = tumour, 0 = background. A TensorFlow U-net model adapted from https://github.com/zhixuhao/unet was trained on a dataset of 595 paired images with masks (70% training and 30% validation) for 7 epochs until the model began to overfit as indicated by the training accuracy exceeding the validation accuracy without improving loss.

#### Image segmentation and tracking

Images of tumour fluorescence were binned and resized to 512 × 512 px using custom FIJI scripts. Initial segmentation guesses were generated by applying the trained U-net using TensorFlow. Custom FIJI scripts were used to un-bin tumour segmentation to restore the original size and resolution, enable manual review and editing of all tumour segmentation masks, and manually track tumours through multiple timepoints. If a tumour was no longer detectable during the course of an experiment, it was designated a complete responder. If a tumour decreased from its maximum size by 20% or more, it was designated a partial responder. The remaining tumours were designated as stable disease and progressing disease.

#### T cell quantification

Custom FIJI scripts were used to identify the centroid and Feret diameter of each tumour region of interest and determine the median radial fluorescence profile of the T cell fluorescence channel. Custom Python scripts were used to determine the overall median T cell fluorescence intensity and categorize radial fluorescence profiles as desert, excluded or inflamed. Tumours were classified as excluded or inflamed using a ratiometric cut-off. If the radial profile within the inner 25% of the tumour was consistently greater than 60% of the maximum fluorescence for that tumour, it was designated as inflamed. If the radial profile within the inner 25% of the tumour was consistently less than 40% of the maximum fluorescence for that tumour, it was designated as excluded. A tumour was designated a desert if the individual tumour’s median T cell fluorescence intensity was less than the 25 percentile of median T cell fluorescence for all tumours measured on the first imaging day and the radial profile did not indicate an excluded pattern as described above. If a tumour showed an excluded profile based on the ratiometric criteria, but the T cell intensity at the core of the tumour (inner 25%) was greater than the median T cell intensity for all inflamed tumours, it was reclassified as inflamed. If a tumour did not meet the above ratiometric cut-offs, the phenotype determined the previous day was propagated forward until the next definitive classification.

#### Clustering and Markov analysis

Custom Python scripts were used to assign an integer value to the T cell phenotype classification with 1 = desert, 2 = excluded, 3 = inflamed. If the mouse was euthanized, the remaining timepoints were assigned a value of 0. If the tumour resolved, the remaining timepoints were assigned a value of 5. Tumour trajectories were ordered by hierarchical clustering of tumour phenotype lists. Subsequent heat maps for tumour area, median T cell infiltration and tumour growth were ordered according to this phenotype clustering. Transition state matrices for Markov analysis were generated using custom Python scripts. To assess significance, phenotype states were randomized ten times using Python random.shuffle() and new transition state matrices were calculated.

#### T cell trajectory analysis

Custom python scripts were used to calculate the infiltration ratio where the numerator is the median intensity at the core of the tumour (inner 25%), and the denominator represents the maximum value of the median radial profile. These values were plotted relative to the total T cell abundance and compared between tumours across timepoints.

### Whole-exome sequencing analyses

To rule out the possibility that TIPs could be explained by pre-existing major subclones within the KPP-eGFP cell line, the tumour cell line genetic heterogeneity was assessed using whole-exome sequencing before implantation and 3 weeks after implantation. A spleen sample from the wild-type mouse strain used in the experiments was used as the reference matched normal genome to perform variant calling in tumour cells. The analysis after tumour implantation was restricted to the tumour variants detected in the KPP-eGFP cell line to alleviate the effect of any potential genetic variability within mice captured from the different TME components of the different biopsies.

Exome capture libraries were sequenced on the HiSeq 2500 (Illumina) system to generate 2 × 75 bp paired-end data; from the sequenced reads, variants were called using the following workflow. Sequencing reads were mapped to the UCSC mouse genome (GRCm38) using BWA software^31^ set to the default parameters. Local realignment, duplicate marking and raw variant calling were performed according to GATK best practices^32^. Somatic variant calling on tumour and its matched normal BAM file was performed using Strelka^33^.

The resulting variants can be interpreted as a description of the genetic differences in the exome present in the KPP-eGFP cell line with regard to the host mouse genome. This measurement coarse grains over the clonal structure of the population; nevertheless, the inspection of the resulting variant allele frequency histogram can inform of potential subclones. For the preimplantation cell line samples, the variant allele frequency was summarized with a histogram. No major subclones were observed in these representations; however, as expected, there was evidence of minor variants. These are potentially neutral minor subclones, which are unavoidable during culturing practices such as an expansion phase after single-cell subcloning. To demonstrate that this low-frequency tail of minor subclones conformed to a neutral accumulation of mutants in the population, the left tail of the histograms was fit to a neutral model using the R package neutralitytestr (https://github.com/marcjwilliams1/neutralitytestr). For the tumour samples after implantation, some potential variants were detected as they were consistently uniquely present in either the immune-inflamed, the immune-excluded or the immune-desert tumours. To exclude having missed the variants due to the algorithm settings and the sequencing depth of each sample, the problematic variants were manually curated by inspecting the reads mapping to the regions in which the variants were detected on the remaining samples using the Integrative Genome Viewer. We therefore confirmed that, out of 48 potentially explanatory variants across immune phenotypes, all but one was detected on the other samples. The missing variant was a very-low-frequency variant that could be explained by not reaching the detection limit in the other tumour samples.

### STAMP microtumour bulk RNA-seq analysis

Each RNA-seq experiment was analysed using HTSeqGenie pipeline in BioConductor^34^ as follows: first, reads with low nucleotide qualities (70% of bases with quality < 23) or matches to rRNA and adapter sequences were removed. The remaining reads were aligned to the mouse reference genome GRCm38.p5 using GSNAP (v.2013-10-10-v2), allowing a maximum of two mismatches per 75 base sequence (parameters: ‘-M2 -n 10 -B 2 -i 1 -N 1 -w 200000 -E 1 --pairmax-rna = 200000 --clip-overlap’). Transcript annotation was based on the Gencode genes database^35^. To quantify gene expression levels, the number of reads mapping unambiguously to the exons of each gene was calculated.

The resulting count matrix was analysed in R (v.4.0.5; 31 March 2021) using the edgeR package (v.3.32.1). The count matrix was filtered to remove low-expressed genes by keeping the features with at least 0.2 counts per million (cpm) in more than the minimum number of samples of the experimental group given the design factor being analysed. The resulting filtered count matrix was then log-transformed and TMM-normalized with edgeR::cpm(log=T) and edgeR::calcNormFactors(method = “TMM”) to perform exploratory analyses. The matrix was further filtered to the most variable genes selected using projection score^36^ to focus on the major contributors to the variance in transcriptional state. This matrix was then scaled using base:scale() for posterior exploration. A heat map of the resulting data matrix annotated by Gene Ontology (GO) terms was constructed for preliminary interpretation by first clustering the genes and then running enrichment analysis using clusterProfiler (v.3.18.1) to select the most significant GO terms associated with said clusters (our script also allowed us to use WikiPathways and KEGG). To understand the general clustering of the samples in transcriptional state, dimensionality reduction of the scaled matrix using principal component analysis was performed using PCAtools (https://github.com/kevinblighe/PCAtools; v.2.5.15).

Differential expression analyses were conducted using limma (v.3.46.0)^37^. To detect signature differences among immune phenotypes, our contrasts compared the immune phenotype at hand with the average of the other two. Volcano plots were constructed using the package Enhanced-Volcano (https://github.com/kevinblighe/EnhancedVolcano; v.1.8.0). Gene set enrichment analysis was performed on the log-transformed fold change given by the differential expression contrasts using the GSEA function from ClusterProfiler on the Hallmark Gene Set Collection^38^. Pathways were considered to be significant if their false-discovery-rate-adjusted *P* value was less than 0.2. The same analysis was performed on the clinical trial RNA-seq data. Heat maps of the normalized enrichment score were constructed for those significant pathways. For particular pathways that were further investigated in detail, samples were scored using the first principal component of the expression matrix of the genes composed the signature multiplied by the sign of the correlation of the component with the sample average expression of all the signature genes. For the time-series experiment, the type I interferon signature was extracted from the Hallmark Gene Set Collection.

### scRNA-seq analysis of STAMP microtumours

scRNA-seq fastq files were processed using CellRanger count (CellRanger v.4.0.2 from 10x Genomics) using a custom reference based on the mouse genome GRCm38 with GENCODE^35^ gene models together with the sequences for the transgenes used in our experiments (tdTomatoand GFP). Similarly, TCR sequencing data were processed using CellRanger vdj.

Analyses of the count matrices were conducted in R (v.4.0.5; 31 March 2021) using Seurat^39^ (v.4.0.4). Only high-quality cells were retained for the posterior analysis; more concretely, we retained the cells with more than 300 hundred genes detected, more than 1,000 unique molecular identifiers and less than 10% mitochondrial reads. To simplify our analyses, tdTomato^+^ cells (adoptive T cells) and GFP^+^ cells (KPP tumour cells) were separated and clustered independently. The remaining cells were clustered to construct an atlas for stamp tumours. In both cases, the clustering and identification of cell populations proceeded following Seurat’s SCTransform pipeline. First, data were normalized using Seurat::SCTransform() with cell cycle regression and batch correction. The number of principal components retained for clustering was then calculated using the talus plot^40^. With these retained components, we then computed a UMAP embedding and the neighbours for posterior clustering. Several clustering resolutions were calculated and a directed tree was constructed reflecting the hierarchical relationships of the new clusters after increasing the resolution^41^. A resolution was considered to be optimal if it did not break the hierarchical structure of the said tree. The main clusters were identified by means of expression markers of known biology. Then, for each major cell cluster, a second run of clustering was performed by iterating the aforementioned pipeline. Notably, this step enabled us to identify heterogeneity within our cell populations and further remove low-quality cells. Markers for each cluster were identified using the Seurat::FindAllMarkers() method with the default parameters, comparing all cells in a particular cluster to the rest of cells and accessing significantly differential gene expression using Wilcoxon’s rank-sum tests and Benjamini–Hochberg correction for multiple testing.

We used Seurat’s plotting functionalities for most plots. Maker heat maps were generated using the package ComplexHeatmap using results from the Seurat::AverageExpression() function as the input after scaling to relative expression per gene using the *z-*score. Differential expression analyses were performed using Seurat::FindMarkers(), with batch as a latent variable and the negative binomial test. We reported the significant gene log-transformed fold change values as a *z*-score-scaled matrix using ComplexHeatmap. Clonotype analysis and integration with Seurat were performed using the scRepertoire package (v.1.0.0). Clonotypes were called according to their TCR amino acid sequence. Gene set enrichment analysis of the top seven dominant clonotypes was performed on the log-transformed fold change values from Seurat::FindMarkers(), comparing the inflamed to the excluded phenotypes using gseGO() function of the ClusterProfiler R Package on the CC Ontology collection.

To demonstrate the lack of deterministic correlation between the transcriptional heterogeneity of the KPP-eGFP cell line in vitro and the immune phenotypes in vivo, paired samples of the cell line before and after implantation were single-cell sequenced. Tumours were cell-hashed with antibodies and were then pooled before sequencing. The samples were demultiplexed after centred log ratio transformation normalization on the hashing antibody counts using Seurat::HTODemux() and doublets and negative cells were removed. Both the cell-hashed samples and the in vitro preimplantation single-cell objects were then processed and clustered according to the same pipeline as for the STAMP atlas. Finally, preimplantation in vitro clusters were connected to in vivo clusters with labels predicted from running SingleR (v.1.8.1) on the in vivo cells with in vitro clusters as a reference. The predicted labels were then used to construct an alluvial plot using ggalluvial (v.0.12.3).

### Statistical analysis and reproducibility

Statistical analysis was performed using two-tailed *t*-tests, Mann–Whitney *U*-tests or log-rank tests as indicated in the figure legends. *P* < 0.05 was considered to be statistically significant. All box and whisker plots demarcate the median (centre line), 75th and 25th percentiles (upper and lower bounds, respectively), and the minimum and maximum values excluding points determined to be outliers by exceeding 1.5× the interquartile range (whiskers). The bar graphs represent the mean ± s.d. or mean ± s.e.m. as indicated. Mice, tumour and cell numbers per condition are provided in the figure legends. Statistical analysis was performed using GraphPad Prism (v.9.4.1) or Python (v.3.10.3) using SciPy (v.1.8.0). Flow cytometry analysis was performed using FlowJo (v.10). The imaging experiments shown in Figs. 1a, 2a,f,g and 3d,h and Extended Data Figs. 1a–c, 2b,e,g, 3c,e,f, 6a,b,f,h,l,m and 9a,e depict representative images and the quantifications are the aggregate of biological replicates as indicated. Mouse experiments were reliably reproduced. Experiments were replicated independently at least twice unless otherwise stated in the legend. No statistical methods were used to determine sample size. Mice were randomized before treatment. Investigators were not blinded to allocation. Treatment experiments were performed blinded for automated high throughput analysis. NGS and Flow cytometry analysis on tumour biopsies were not performed blinded owing to the needs to track single tumour biopsies and pool them by similar features (genotype of the mouse, immune phenotypes, time). For all the experiments, analysis was objective.

### Figure preparation

All data were assembled into figures with Adobe Illustrator 2022. Figs. 1a, 2c and 3g were created using BioRender (https://biorender.com/). R plots used native plotting capabilities of the aforementioned packages together with ggplot2 (v.3.3.5), ggpubr (v.0.4.0) and ComplexHeatmap^42^ (v.2.6.2) packages.

### Reporting summary

Further information on research design is available in the Nature Portfolio Reporting Summary linked to this article.

## Online content

Any methods, additional references, Nature Portfolio reporting summaries, source data, extended data, supplementary information, acknowledgements, peer review information; details of author contributions and competing interests; and statements of data and code availability are available at 10.1038/s41586-023-06132-2.

## Supplementary information

Supplementary Information Supplementary Figs. 1–5.

Reporting Summary

Supplementary Table 1 Differentially expressed genes (*q* ≤ 0.05) between individual STAMP tumours of different immune phenotypes (inflamed, desert, excluded) (related to Extended Data Fig. 3a,b).

Supplementary Table 2 Normalized enrichment scores for all pathways that show significance in at least one comparison between the ICON 7 and IMvigor210 human clinical trials and STAMP mouse tumours—inflamed, excluded and desert STAMP tumours share common gene expression programs with clinical tumours of analogous immune phenotype (related to Fig. 2b).

Supplementary Table 3 Top 10 marker genes defining T cell subsets in STAMP microtumours (related to Fig. 2c,d and Extended Data Fig. 5a–c,f).

Supplementary Table 4 Differentially expressed genes by T cell subsets in inflamed versus excluded STAMP microtumours (related to Extended Data Fig. 5g).

Supplementary Table 5 Top10 marker genes defining myeloid cell subsets in STAMP microtumours (related to Fig.3a and Extended Data Fig. 7b,c).

Supplementary Table 6 Top 10 marker genes defining fibroblast subsets in STAMP microtumours (related to Fig.3f and Extended Data Fig. 8a–d).

Supplementary Table 7 Clinical summary (related to Extended Data Fig. 9a).

Supplementary Table 8 Markov probabilities of the transition between immune phenotypes (related to Fig. 4d).

Supplementary Video 1 Example of live in vivo two-photon imaging of an individual KPP BFP^+^ STAMP tumour showing the migration of TdTomato^+^ OTI T cells and WT GFP^+^ T cells.

Supplementary Video 2 Confocal imaging followed by 3D reconstruction of a whole cleared lung showing KPP-GFP^+^ tumour foci and TdTomato^+^CD3^+^ T cells 8 days after i.v. transfer (related to Extended Data Fig. 3f).

Supplementary Video 3 Confocal live imaging showing low Ca^2+^ flashes (green) in a GCaMP6^+^HER2^+^ KPP organoid, co-cultured with T cells (magenta) and propidium iodide (Red) (related to Extended Data Fig. 6a).

Supplementary Video 4 Confocal live imaging showing high Ca^2+^ flashes (green) in a GCaMP6^+^HER2^+^ KPP organoid, co-cultured with T cells (magenta) and TDB (anti-HER2/anti-CD3) to force tumour cell killing by T cells (positive control). Propidium iodide entry (red) reveals tumour cell death (related to Extended Data Fig. 6b).

Supplementary Video 5 In vivo two-photon live imaging showing absence of Ca^2+^ flashes in GCaMP6^+^BFP^+^HER2^+^ KPP tumours implanted in nude mice (Related to Extended Data Fig. 6h).

Supplementary Video 6 In vivo two-photon live imaging showing some Ca^2+^ flashes in GCaMP6^+^BFP^+^HER2^+^ KPP tumours implanted into nude mouse reconstituted with TdTomato^+^ T cells (related to Extended Data Fig. 6f,h).

Supplementary Video 7 In vivo two-photon live imaging showing high level of Ca^2+^ flashes in GCaMP6^+^BFP^+^HER2^+^KPP tumours implanted in nude mice reconstituted with TdTomato^+^ T cells and treated with TDB (anti-HER2/anti-CD3) to force tumour cell killing by T cells (related to Extended Data Fig. 6f).

Supplementary Video 8 Epifluorescence live imaging of an immune inflamed tumour showing high Ca^2+^ flashes in GCaMP6^+^ KPP tumour cells implanted in nude mice reconstituted with TdTomato^+^ T cells (related to Extended Data Fig. 3l).

Supplementary Video 9 Epifluorescence live imaging of an immune-excluded tumour showing low Ca^2+^ flashes (green) in GCaMP6^+^ KPP tumour cells implanted in nude mice reconstituted with TdTomato^+^ T cells (related to Extended Data Fig. 3m).

## Data Availability

All mouse sequencing data are publicly available at the Gene Expression Omnibus (GSE222231) (whole-exome sequencing, bulk RNA-seq, scRNA-seq). The ICON7 and IMvigor210 human datasets have been previously published^11,13^ and deposited. IMvigor210 RNA-seq data were deposited to the European Genome–Phenome Archive under accession number EGAS00001002556. ICON7 raw RNA-seq and clinical data were deposited to the European Genome–Phenome Archive under accession number EGAS00001003487. Source data are provided with this paper.

## Source data

Source Data Fig. 1
Source Data Fig. 2
Source Data Fig. 3
Source Data Fig. 4
Source Data Extended Data Fig. 1
Source Data Extended Data Fig. 2
Source Data Extended Data Fig. 3
Source Data Extended Data Fig. 5
Source Data Extended Data Fig. 6
Source Data Extended Data Fig. 7
Source Data Extended Data Fig. 8
Source Data Extended Data Fig. 9
Source Data Extended Data Fig. 10

## Code availability

The STAMP image segmentation and analysis pipeline was developed using custom scripts in FIJI and Python and is available at GitHub (https://github.com/kcarbone/STAMP).
