# Supplementary material for: In situ tumour arrays reveal early environmental control of cancer immunity
Source: Nature. 2023 May 31;618(7966):827–33. doi: 10.1038/s41586-023-06132-2 (PMC10284705; doi:10.1038/s41586-023-06132-2)
Supplement: Supplementary file 1 — Supplementary Figs. 1–5. [file 41586_2023_6132_MOESM1_ESM.pdf]

nature portfolio

https://doi.org/10.1038/s41586-023-06132-2

Supplementary information

In situ tumour arrays reveal early environmental control of cancer immunity

In the format provided by the authors and unedited

***In situ* tumor arrays reveal early environmental control of cancer immunity**

Guadalupe Ortiz-Muñoz[1]*, Markus Brown[1]*, Catherine B. Carbone[1]*, Ximo Pechuan-Jorge[1]*, Vincent Rouilly[1], Henrik Lindberg[1], Alex T. Ritter[1], Gautham Raghupathi[10], Qianbo Sun[1], Tess Nicotra[1], Shreya R. Mantri[1], Angela Yang[4], Jonas Doerr[2], Deepti Nagarkar[1], Spyros Darmanis[4], Benjamin Haley[2], Sanjeev Mariathasan[3], Yulei Wang[3], Carlos Gomez-Roca[6], Carlos Eduardo de Andrea[7], David Spigel[8], Thomas Wu[5], Lelia Delamarre[1], Johannes Schöneberg[9], Zora Modrusan[4], Richard Price[3], Shannon J. Turley[1], Ira Mellman[1], Christine Moussion[1]†

[1]Department of Cancer Immunology, Genentech, Inc., South San Francisco, CA, USA
[2]Department of Molecular Biology, Genentech, Inc., South San Francisco, CA, USA
[3]Department of Oncology Biomarker Development, Genentech, Inc., South San Francisco, CA, USA
[4]Department of Microchemistry, Proteomics and Lipidomics, Genentech, Inc., South San Francisco, CA
[5]Department of Bioinformatics and Computational Biology Genentech, Inc., South San Francisco, CA
[6]IUCT, Institut Universitaire du Cancer de Toulouse, France
[7]Department of Pathology, Clínica Universidad de Navarra, Pamplona, Spain
[8]Sarah Cannon Research Institute, Nashville, USA
[9]Departments of Pharmacology and Chemistry & Biochemistry, UCSD, San Diego, CA, USA
[10]Department of Computer Science, Stanford University, Stanford, CA, USA
* These authors contributed equally to this work
† Email: moussion.christine@gene.com

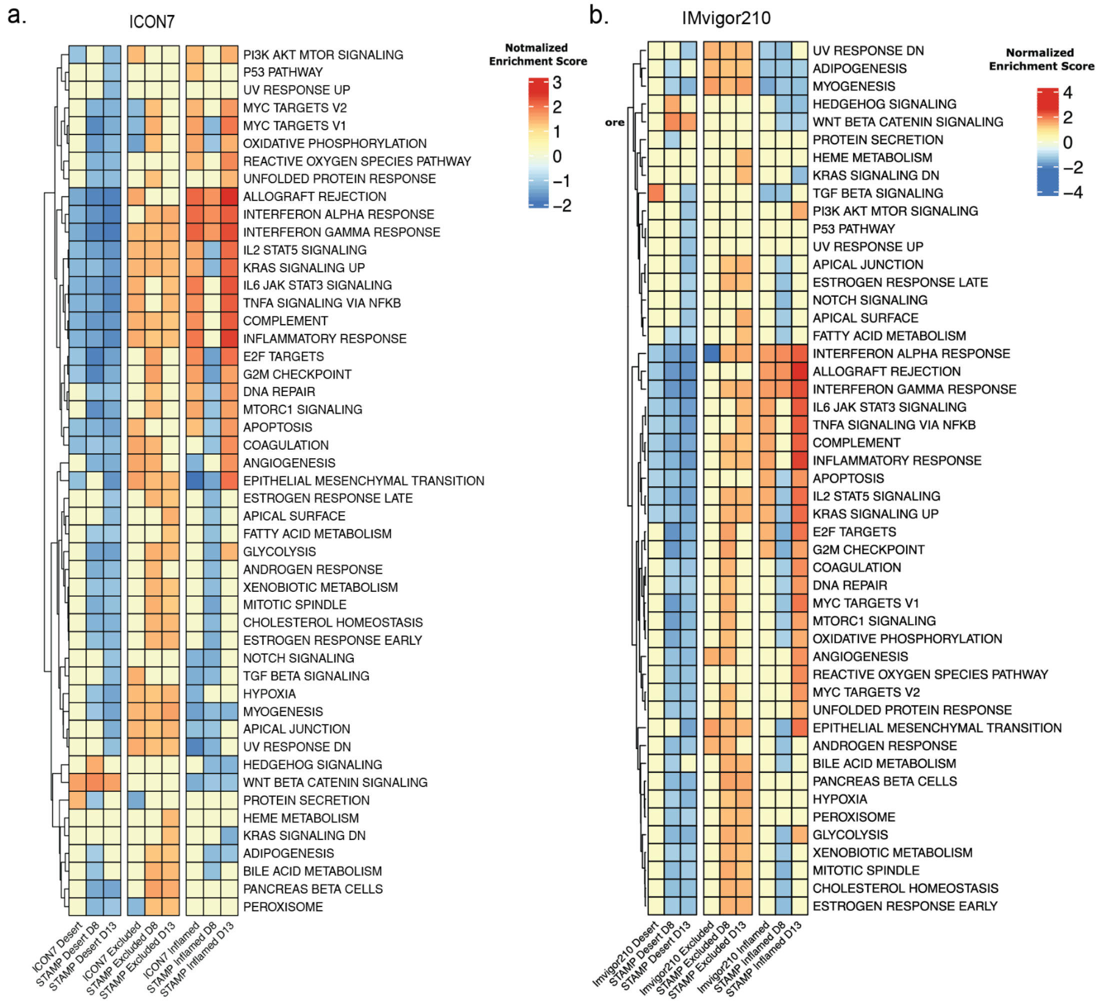

Supplementary Data.1 | Inflamed, excluded and desert microtumors share gene expression programs with analogous clinical tumors

a, Heatmap comparing the normalized enrichment scores for all pathways that show significance in at least one comparison of the different immune phenotypes of the ICON 7 human clinical trial and STAMP mouse tumors. **b,** Heatmap comparing the normalized enrichment scores for all pathways that show significance in at least one comparison of the different immune phenotypes of the IMvigor 210 human clinical trial and STAMP mouse tumors. Normalized Enrichment Scores were reported using clusterProfiler::GSEA with fdr p-value adjustment method and considering significant those with p-adjusted < 0.2.

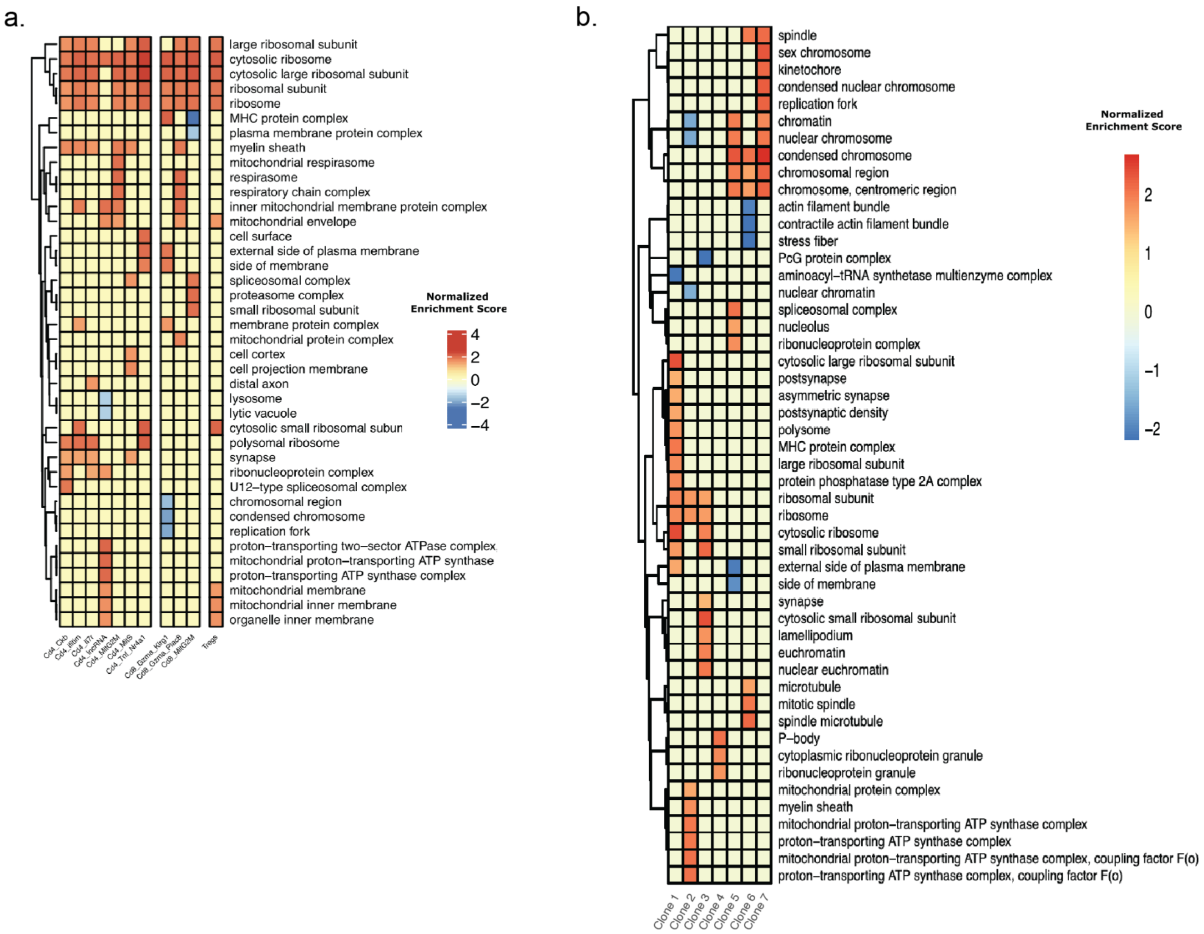

Supplementary Data.2 | Gene set enrichment analysis of T cell subsets and clonotypes comparing inflamed and excluded microtumors

a, Gene set enrichment analysis for each T cell subcluster comparing inflamed versus excluded tumors. **b**, Gene set enrichment analysis for 7 most abundant T cell clonotypes comparing inflamed versus excluded tumors. Normalized Enrichment Scores were reported using clusterProfiler::GSEA with fdr p-value adjustment method and considering significant those with p-adjusted < 0.2.

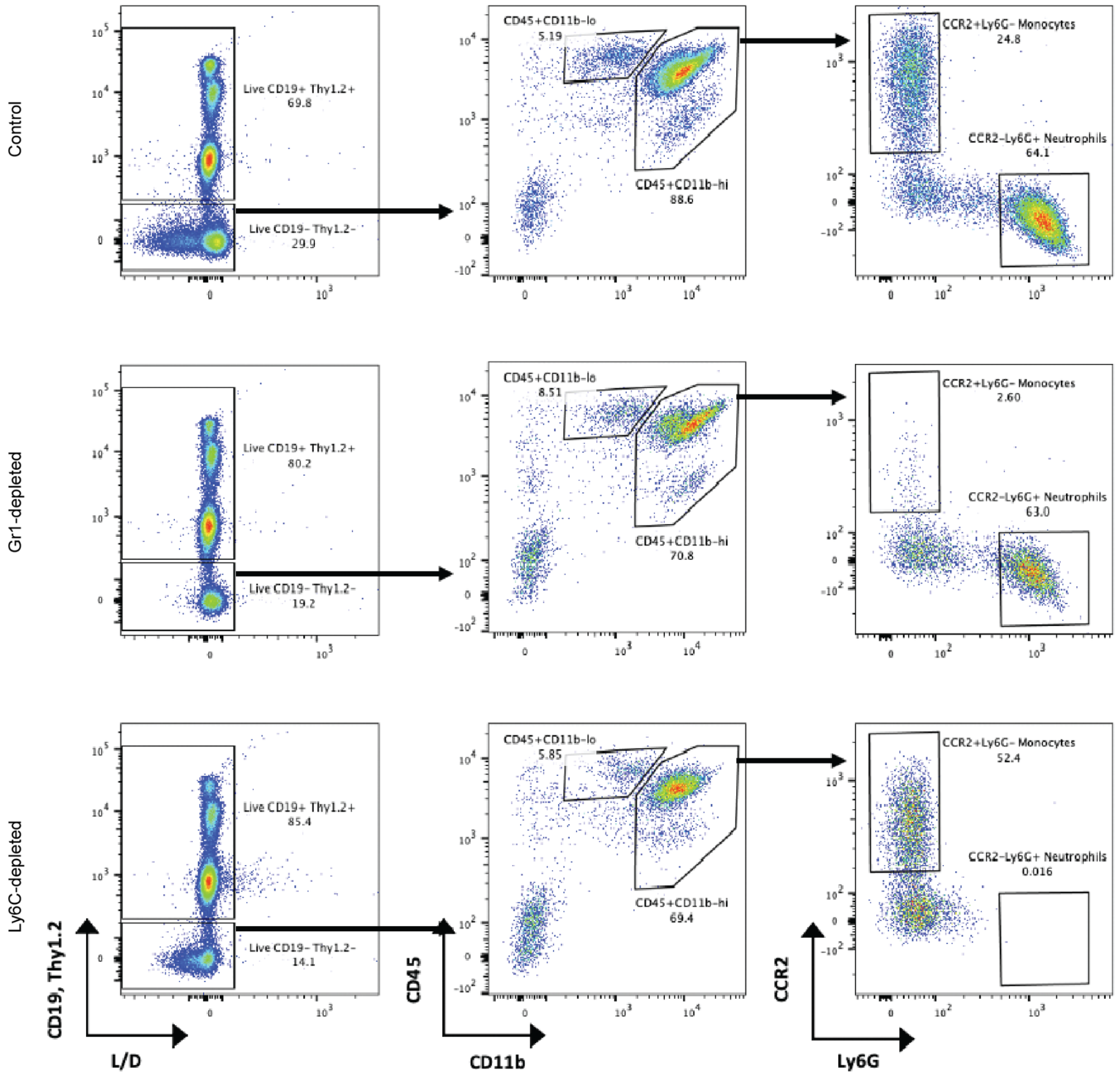

Supplementary Data.3 | Gating strategy for neutrophil and myeloid depletion experiments.
Gating strategy and representative flow cytometry plots showing efficient neutrophil and monocyte depletion with Gr1 and Ly6C antibodies prior to STAMP implantation. n=5 animals per group.

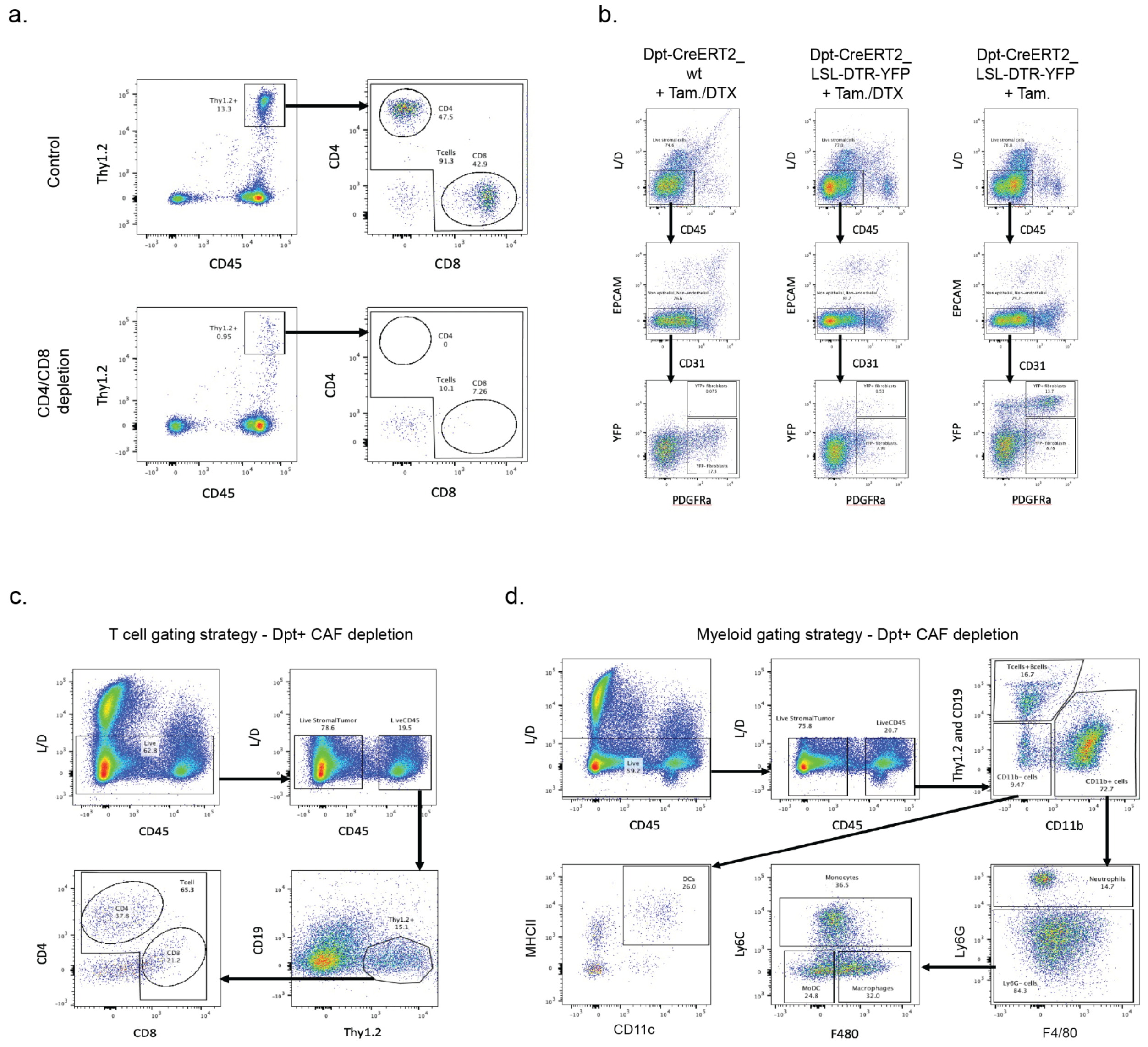

Supplementary Data.4 | Gating strategy for Dpt+ fibroblasts depletion experiment: a, Gating strategy and representative flow cytometry plots showing efficient T cell depletion with CD4 and CD8 depleting antibodies prior to adoptive transfer of tdTomato positive T cells to enable characterization of desert, inflamed and excluded tumors by fluorescence microscopy. n=5 mice per group. **b,** Gating strategy and representative flow cytometry plots showing tamoxifen (Tam.) induction of YFP expression in skin fibroblasts and diphtheria toxin (DTX) induced ablation of YFP-expressing skin fibroblasts as pertaining to Fig.3b. **c**, Flow cytometry gating strategy for T cell profiling in KPP-EGFP STAMP tumors of Dpt+ skin fibroblast-depleted mice. **d,** Flow cytometry gating strategy for myeloid cell profiling in KPP-EGFP STAMP tumors of Dpt+ skin fibroblast-depleted mice. n≧5 mice per group.

a.

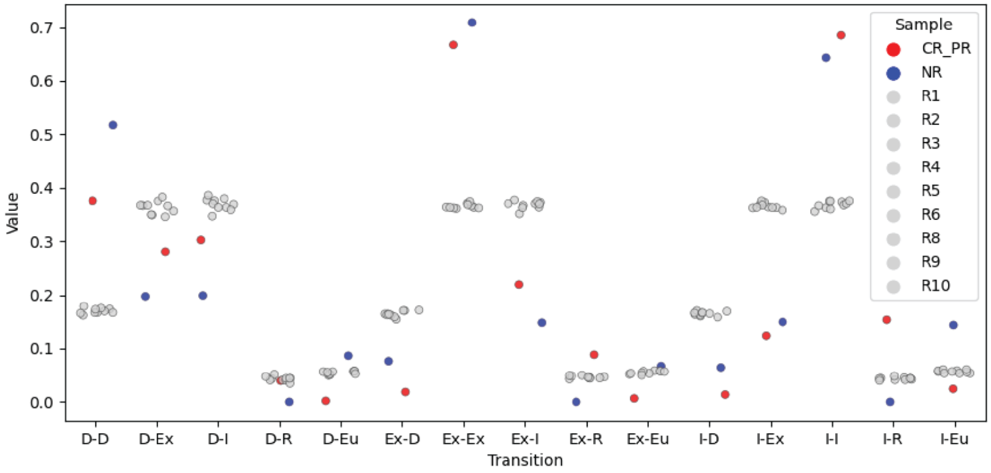

Supplementary Data.5 | Markov chain transition matrix compared to 10 matrices bootstrapped from the same input data

a, Markov chain transition matrix for data represented in **Fig. 4g** compared to 10 matrices bootstrapped from the same input data. KPP-EGFP STAMP tumor arrays were implanted in Rag-2-deficient animals reconstituted with tdTomato+ T cells and and treated at day 2 post-implantation with isotype control antibodies (n=554 tumors, 9 animals) or a combination of anti–PD-L1 with anti–TGF-β (n=642 tumors, 11 animals per group).
