# Supplementary material for: In situ tumour arrays reveal early environmental control of cancer immunity
Source: Nature. 2023 May 31;618(7966):827–33. doi: 10.1038/s41586-023-06132-2 (PMC10284705; doi:10.1038/s41586-023-06132-2)
Supplement: Supplementary file 2 — Reporting Summary [file 41586_2023_6132_MOESM2_ESM.pdf]

nature portfolio

Corresponding author(s): Christine Moussion

Last updated by author(s): Feb 6, 2023

Reporting Summary

Nature Portfolio wishes to improve the reproducibility of the work that we publish. This form provides structure for consistency and transparency in reporting. For further information on Nature Portfolio policies, see our Editorial Policies and the Editorial Policy Checklist.

Statistics

For all statistical analyses, confirm that the following items are present in the figure legend, table legend, main text, or Methods section.

n/a	Confirmed	
☐	☒	The exact sample size (*n*) for each experimental group/condition, given as a discrete number and unit of measurement
☐	☒	A statement on whether measurements were taken from distinct samples or whether the same sample was measured repeatedly
☐	☒	The statistical test(s) used AND whether they are one- or two-sided<br>*Only common tests should be described solely by name; describe more complex techniques in the Methods section.*
☒	☐	A description of all covariates tested
☐	☒	A description of any assumptions or corrections, such as tests of normality and adjustment for multiple comparisons
☐	☒	A full description of the statistical parameters including central tendency (e.g. means) or other basic estimates (e.g. regression coefficient) AND variation (e.g. standard deviation) or associated estimates of uncertainty (e.g. confidence intervals)
☐	☒	For null hypothesis testing, the test statistic (e.g. *F*, *t*, *r*) with confidence intervals, effect sizes, degrees of freedom and *P* value noted<br>*Give P values as exact values whenever suitable.*
☒	☐	For Bayesian analysis, information on the choice of priors and Markov chain Monte Carlo settings
☒	☐	For hierarchical and complex designs, identification of the appropriate level for tests and full reporting of outcomes
☐	☒	Estimates of effect sizes (e.g. Cohen's *d*, Pearson's *r*), indicating how they were calculated

Our web collection on statistics for biologists contains articles on many of the points above.

Software and code

Policy information about availability of computer code

Data collection	BD Biosciences FACS DIVA software v. 7, Leica Application Suite X (LAS X)
Data analysis	BD Biosciences FlowJo v. 10.8.1, GraphPad Prism v.9.4.1, Python v.3.10.3, SciPy v.1.8.0, R version 4.0.5, neutralitytestr v. 0.0.3, edgeR R package v. 3.32.1, clusterProfiler v. 3.18.1, PCAtools v. 2.6.0 , limma v. 3.46.0, EnhancedVolcano v. 1.12.0, EmbolcallRNAseq v. 0.1.0, CellRanger 4.0.2, Seurat v. 4.0.4, scRepertoire v. 1.0.0, ggalluvial v. 0.12.3, SingleR v. 1.8.1, tidyverse v. 1.3.2.<br><br>NGS: Analyses were perfommed using custom scripts (https://github.roche.com/pechuanj/StampAnalyses), HTSeqGenie,BWA,GATK,Strelka GSNAP ,cellranger and the R packages: EnhancedVolcano, factorextra,Seurat,voom,limma,tidyverse,ClusterProfiler,ComplexHeatmap,scRepertoire and neutralitytestr.<br>Image segmentation and analysis pipeline was developed using custom scripts in FIJI and Python.

For manuscripts utilizing custom algorithms or software that are central to the research but not yet described in published literature, software must be made available to editors and reviewers. We strongly encourage code deposition in a community repository (e.g. GitHub). See the Nature Portfolio guidelines for submitting code & software for further information.

Data

Policy information about availability of data

All manuscripts must include a data availability statement. This statement should provide the following information, where applicable:

- Accession codes, unique identifiers, or web links for publicly available datasets
- A description of any restrictions on data availability
- For clinical datasets or third party data, please ensure that the statement adheres to our policy

Whole exome sequencing, bulk RNA sequencing and single cell RNA sequencing data of STAMP tumors have been deposited on the Gene Expression Omnibus (GSE222231).
Image segmentation and analysis pipeline was developed using custom scripts in FIJI and Python and is hosted on GitHub at https://github.com/kcarbone/STAMP.

Human research participants

Policy information about studies involving human research participants and Sex and Gender in Research.

Reporting on sex and gender

The sex of all patients in the study was collected by consenting physician and recorded as part of the demographic data in the CRF. No consideration to enrolling one sex or the other was given. Findings are analyzed as case-studies and are as such not generalizable to one sex or another.

Population characteristics

We are presenting data from cancer patients from several indications:
- metastatic urothelial carcinoma for IMvigor210 clinical trial, previously published as Mariathasan et al, Nature 2018
- ovarian cancer for ICON7 clinical trial, previously published as Desbois et al, Nature Comm 2020
- imCORE study: the patient population included adults with metastatic cancer including melanoma, squamous cell carcinoma of the head/neck and non-small cell lung cancer.

-For ICON7 study, three hundred seventy treatment-naive patient samples with epithelial ovarian cancer from mixed histology were collected from the Phase III ICON7 clinical trial, and the clinical characteristics of these patients are summarized in this Table:

CARBOPLATIN PLUS PACLITAXEL CHEMOTHERAPY / CARBOPLATIN PLUS PACLITAXEL CHEMOTHERAPY PLUS BEVACIZUMAB
n 182 / 188
Histology % (n) / % (n)
Serous 71 (130) / 69 (130)
Clear cell 10 (18) / 13 (25)
Endometroid 9 (16) / 5 (9)
Mucinous 2 (3) / 1 (2)
Serous/ClearCell/Endometroid/Mucinous/Other Mixed 5 (9) / 9 (16)
Endometroid/Clear cell/Mixed 1 (1) / 2 (3)
PapillaryCystoadenocarcinoma/Unclassified/Undififfentated/ Adenocarcinoma 3 (5) / 2 (3)
Original cancer % (n) / % (n)
Ovary (epithelial) 90 (164) / 88 (165)
Fallopian tube 2 (4) / 3 (5)
Primary peritoneum 5 (10) / 7 (14)
Mixed 2 (4) / 2 (4)
Age Group % (n) / % (n)
18-39yr 4 (8) / 3 (5)
40-64yr 72 (131) / 76 (144)
>=65 23 (43) / 21 (39)
FIGO Stage % (n) / % (n)
Stage I 8 (14) / 8 (15)
Stage II 11 (20) / 14 (26)
Stage III 72 (131) / 68 (127)
Stage IV 9 (17) / 11 (20)
Grade % (n) / % (n)
1 5 (10) / 4 (8)
2 16 (30) / 16 (31)
3 77 (141) / 79 (148)
unknown 0 (1) / 1 (1)
Platinum Sensitivity % (n) / % (n)
Sensitive 49 (89) / 62 (117)
Intermediate 19 (35) / 20 (37)
Resistant 27 (50) / 16 (30)
Refractory 1 (2) / 0 (0)
NA 3 (6) / 2 (4)
Baseline CA-125 Category % (n) / % (n)
< 2x ULN 49 (89) / 39 (74)
>= 2x ULN 50 (92) / 60 (112)
NA 1 (1) / 1 (2)

- For IMvigor210 trial: IMvigor210 RNASeq data has been previously submitted to EGA as part of the Mariathasan et al, Nature 2018 manuscript. The metadata associated with that includes gender, cohort, ORR, IC and TC status, CIT phenotypes, TMB as well as Lund and TCGA subtypes. Additional meta data cannot be added to this submission due the fact that the patients have been de-identified per Roche data sharing policies, in line with evolving international privacy laws.

- For imCORE study: Clinical characteristics of patients are summarized in the Supplementary Table 7 of this manuscript

Recruitment

This information is available for ICON7 and IMvigor210 in their previous publications.
For imCORE trial, patients were recruited by participating institutions if eligibility criteria (including clinical benefit from checkpoint inhibition and biopsies were available before and after treatment from the same tissue) were met. No knowledge of immunophenotype was known at the time of recruitment, thus limiting potential bias.

Ethics oversight

IMvigor210 trial: The study was approved by the independent review board at each participating site and was done in full conformance of the provisions of the Declaration of Helsinki and Good Clinical Practice Guidelines.
Approval from the Institutional Ethics Committee (IEC) or the Institutional Review Board (IRB) was obtained before study start and was documented in a letter to the investigator specifying the date on which the committee met and granted the approval.

The ICON7 protocol was compliant with good clinical practice guidelines and the Declaration of Helsinki. Approval by ethics committees was obtained at each clinical site, nationally, or both.

imCORE trial: The study protocol was approved at enrolling institutions and by local ethics committees (Sarah Cannon Research Institute - WIRB; IUCT Oncopole Toulouse, France; Clinica Universidad di Navarra, Spain)

All patients have provided written informed consent.

Note that full information on the approval of the study protocol must also be provided in the manuscript.

Field-specific reporting

Please select the one below that is the best fit for your research. If you are not sure, read the appropriate sections before making your selection.

☒ Life sciences ☐ Behavioural & social sciences ☐ Ecological, evolutionary & environmental sciences

For a reference copy of the document with all sections, see nature.com/documents/nr-reporting-summary-flat.pdf

Life sciences study design

All studies must disclose on these points even when the disclosure is negative.

Sample size

No sample size calculation was conducted for IMvigor210 and imCORE clinical trials.
The sample size for ICON7 was calculated, as previously published, to provide 83% power to detect a 28% change in progression-free survival at 18 months of follow-up and 80% power to identify a 19% improvement in overall survival, each with a two-sided significance level of 5%, allowing for up to 5% non-compliance with protocol.
We reported all tumor RNAseq data that is available from patients in IMvigor210 and ICON7 trials. Not all patients in ICON7, IMvigor210 and imCORE have immunophenotyping data available so we analyzed samples with both matching RNAseq and immunophenotyping data.
In mice experiment: no statistical methods were used to predetermine sample size. because STAMP technology allows high throughput analysis of several hundreds of tumors per condition. The statistical power of STAMP is way superior to the current standard in the field classically analyzing 10 tumors per group.
NGS: For the sequencing analysis, sample sizes were determined by a balance of technical feasibility and statistical power. For the single cell sequencing we were limited by the number of cells present in each individual punch biopsy of microtumor.

Data exclusions

ICON7 and IMvigor210: Patients without tumor immunophenotyping data were excluded from analysis
imCORE trial: Patients without tumor immunophenotyping data or with paired tumor biopsies performed on different lesions were excluded from the analysis.
NGS analysis of microtumors: Quality control was performed to exclude any technical outlier of all the sequencing data. For the Day 13 bulk RNA seq, two samples were excluded on the basis of miss-identification of the tumor immune phenotype as given by aberrant gene expression profiles.

Replication

IMvigor210, ICON7 and imCORE human clinical trials are unique data set and no replication was made. Animal experiments were reliably reproduced. Experiments were replicated independently at least two times unless otherwise stated in the legend.

Randomization

No randomization was conducted for IMvigor210 and imCORE trials. In IMvigor210 Phase II trial, patients were treated with atezolizumab monotherapy. For imCORE trial patients were assigned to a single group and treated with checkpoint inhibitors.
For ICON7, women with ovarian cancer were randomly assigned to carboplatin (area under the curve, 5 or 6) and paclitaxel (175 mg per square meter of body-surface area), given every 3 weeks for 6 cycles, or to this regimen plus bevacizumab (7.5 mg per kilogram of body weight).
Fig4: mice were randomly assigned to the treatment group after tumor implantation. For other mouse experiments, mice were grouped based on genotype.

Blinding	Blinding was not relevant in the IMvigor210 and imCORE trials as all subjects received the same treatment or single Group Assignment.<br>ICON7: double-blinded trial.<br>Mice experiments: STAMP mice treatment experiments have been blinded for automated high throughput analysis. NGS and Flow cytometry analysis on tumor biopsies were not blinded because of the needs to track single tumor biopsies and pool them by similar features (genotype of the animal, immune phenotypes, time...). For all the experiments, analysis was objective.

Reporting for specific materials, systems and methods

We require information from authors about some types of materials, experimental systems and methods used in many studies. Here, indicate whether each material, system or method listed is relevant to your study. If you are not sure if a list item applies to your research, read the appropriate section before selecting a response.

Materials & experimental systems

n/a	Involved in the study
☐	☒ Antibodies
☐	☒ Eukaryotic cell lines
☒	☐ Palaeontology and archaeology
☐	☒ Animals and other organisms
☐	☒ Clinical data
☒	☐ Dual use research of concern

Methods

n/a	Involved in the study
☒	☐ ChIP-seq
☐	☒ Flow cytometry
☒	☐ MRI-based neuroimaging

Antibodies

Antibodies used	Antibodies used:<br>Flow cytometry:<br>Anti-mouse CD19 BV785 (clone 6D5), Biolegend Cat. No. 115543, 1:500<br>Anti-mouse I-A/I-E BV510 (clone M5/114.15.2), Biolegend Cat. No. 107645, 1:200<br>Anti-mouse F4/80 pe dazzle (clone BM8), Biolegend Cat. No.123146, 1:200<br>Anti-mouse/human CD11b PE (clone M1/70), Biolegend Cat. No. 101207, 1:200<br>Anti-mouse Ly6G BV421 (clone 1A8), Biolegend Cat. No.127628, 1:200<br>Anti-mouse Ly6G BV421 (clone 1A8), BD Bioscience Cat. No 562737, 1:200<br>Anti-mouse Ly6C FITC (clone HK1.4), Biolegend Cat. No.128022, 1:200<br>Anti-mouse CD69 BD421 (clone H1.2F3), Biolegend Cat. No.562920, 1:200<br>Anti-mouse CD25 PE-Dazzle (clone PC61), Biolegend Cat. No.102048, 1:200<br>Anti-mouse CD4 BV650 (clone RM4-5), Biolegend Cat. No.100545, 1:200<br>Anti-mouse CD62L BUV737 (clone MEL-14), BD Bioscience Cat. No.612833, 1:200<br>Anti-mouse/human CD44 BV510 (clone IM7), Biolegend Cat. No.103043, 1:200<br>Anti-mouse CD45 BUV395 (clone 30:F11), BD Bioscience Cat. No. 564279, 1:200<br>Anti-mouse CD86 BV711 (clone GL1), BD Bioscience Cat. No. 740688, 1:200<br>Anti-mouse CD11c PE-Cy7 (clone N418), ThermoFisher Scientific Cat. No. 25-0114-82, 1:200<br>Anti-mouse CD8a BV650(clone 53-6.7), BD Bioscience Cat. No. 563234, 1:200<br>Anti-mouse CD3 FITC (clone 17A2), BD Bioscience Cat. No 561798, 1:200<br>Anti-mouse CD19 PE (clone 1D3/CD19), Biolegend Cat. No 152407, 1:200<br>Anti-mouse Thy1.2 PE (clone 30-H12), Biolegend Cat. No 15307, 1:200<br>Anti-mouse CD45 BV605 (clone 30-F11), Biolegend Cat. No 103139, 1:500<br>Anti-mouse CCR2 AF647 (clone SA203G11), Biolegend Cat. No 150603, 1:200<br>Anti-mouse CD11b AF488 (clone M1/70), Biolegend Cat. No 101219, 1:500<br><br>In vivo treatment<br>Anti-mouse PD-L1 (IgG1 clone 6E11), Genentech compound produced in house, 100µg/animal in 100µL<br>Anti-mouse TGF-β (IgG1 clone 1D11), Genentech compound produced in house, 100µg/animal in 100µL<br>Anti-mouse Ly6G/Ly6C (IgG2b clone GR-1), BioXcell Cat. No. BE0075, 300µg/animal in 100µL<br>Anti-mouse Ly6C (IgG2a clone Monts 1), BioXcell Cat. No. BE0203, 300µg/animal in 100µL<br>Anti-mouse CD8b (IgG1 clone Lyt 3.2), BioXcell Cat. No. BE0223, 100µg/animal in 100µL<br>Anti-mouse CD4 (IgG2a clone YTS 117), BioXcell Cat. No. BP0003-3, 100µg/animal in 100µL<br>Anti-human Her2/anti-mouse CD3e TDB, Genentech compound produced in house, 500nM in vitro, 180µg/animal in 100µL<br><br>Isotype controls for in vivo treatment:<br>Anti-mouse gp120 (mouse IgG1; clone 3E5), Genentech, 100µg/animal in 100µL<br>Anti-keyhole limpet hemocyanin (rat IgG2b; clone LTF-2), BioXcell Cat. No. BE0090, 300µg/animal in 100µL<br>Anti-horseradish peroxidase (rat IgG1; clone HRPN), BioXcell Cat. No. BE0088, 100µg/animal in 100µL<br>Anti-trinitrophenol (rat IgG2a; clone 2A3), BioXcell Cat. No. BP0089, 100µg/animal in 100µL
Validation	Validation for commercially available antibodies can be found at manufacturers websites.<br><br>Target fluorochrome manufacturer clone validation:

Anti-mouse CD19 https://www.biolegend.com/en-us/products/brilliant-violet-785-anti-mouse-cd19-antibody-7962?GroupID=BLG10556
Anti-mouse I-A/I-E https://www.biolegend.com/en-us/products/brilliant-violet-785-anti-mouse-cd19-antibody-7962?GroupID=BLG10556Anti-mouse%20I-A/I-E%20BV510%20(clone%20M5/114.15.2),%20Biolegend%20Cat.%20No.%20107645
Anti-mouse F4/80
https://www.biolegend.com/en-us/products/pe-dazzle-594-anti-mouse-f4-80-antibody-10262
Anti-mouse/human CD11b
https://www.biolegend.com/en-us/products/pe-anti-mouse-human-cd11b-antibody-349?GroupID=BLG10552
Anti-mouse Ly6G
https://www.biolegend.com/fr-fr/products/brilliant-violet-421-anti-mouse-ly-6g-antibody-7161
Anti-mouse Ly6C
https://www.biolegend.com/de-at/products/fitc-anti-mouse-ly-6c-antibody-4896
Anti-mouse CD69
https://www.biolegend.com/en-us/products/brilliant-violet-421-anti-mouse-cd69-antibody-7358
Anti-mouse CD25
https://www.biolegend.com/fr-fr/products/pe-dazzle-594-anti-mouse-cd25-antibody-10220
Anti-mouse CD4
https://www.biolegend.com/en-us/products/brilliant-violet-650-anti-mouse-cd4-antibody-7634?GroupID=BLG4745
Anti-mouse CD62L
https://www.bdbiosciences.com/en-us/products/reagents/flow-cytometry-reagents/research-reagents/single-color-antibodies/buv737-rat-anti-mouse-cd62l.612833
Anti-mouse/human CD44
https://www.biolegend.com/fr-fr/search-results/brilliant-violet-510-anti-mouse-human-cd44-antibody-7994
Anti-mouse CD45
https://www.bdbiosciences.com/en-us/products/reagents/flow-cytometry-reagents/research-reagents/single-color-antibodies-ruo/buv395-rat-anti-mouse-cd45.564279
Anti-mouse CD86
https://www.bdbiosciences.com/en-us/products/reagents/flow-cytometry-reagents/research-reagents/single-color-antibodies-ruo/bv711-rat-anti-mouse-cd86.740688
Anti-mouse CD11c
https://www.thermofisher.com/antibody/product/CD11c-Antibody-clone-N418-Monoclonal/25-0114-82
Anti-mouse CD8a
https://www.bdbiosciences.com/en-us/products/reagents/flow-cytometry-reagents/research-reagents/single-color-antibodies-ruo/bv650-rat-anti-mouse-cd8a.563234
Anti-mouse CD3 https://www.bdbiosciences.com/en-us/products/reagents/flow-cytometry-reagents/research-reagents/single-color-antibodies-ruo/fitc-rat-anti-mouse-cd3-molecular-complex.561798

In vivo treatment:
Anti-mouse Ly6G/Ly6C https://bioxcell.com/invivomab-anti-mouse-ly6g-ly6c-gr-1-be0075
Anti-mouse Ly6C https://bioxcell.com/invivomab-anti-mouse-ly6c-be0203
Anti-mouse CD8b https://bioxcell.com/invivomab-anti-mouse-cd8b-lyt-3-2-be0223
Anti-mouse CD4 https://bioxcell.com/invivoplus-anti-mouse-cd4-bp0003-3

Isotype controls for in vivo treatment:
Anti-keyhole limpet hemocyanin https://bioxcell.com/invivomab-rat-igg2b-isotype-control-anti-keyhole-limpet-hemocyanin-be0090
Anti-horseradish peroxidase https://bioxcell.com/invivomab-rat-igg1-isotype-control-anti-horseradish-peroxidase-be0088
Anti-trinitrophenol https://bioxcell.com/invivomab-rat-igg2a-isotype-control-anti-trinitrophenol-be0089

Anti-mouse PD-L1, anti-mouse TGF-β, and anti-mouse gp120 were validated internally at Genentech using in vitro and in vivo (subcutaneous tumors) potency assays in MC38 and EMT6 tumor models.

CD45: Flow cytometric analysis of CD45 expression on mouse splenocytes (website)
CD11c: Flow cytometric analysis of CD11c on expression mouse splenocytes (website)
CD103: Flow cytometric analysis of CD103 expression on mouse splenocytes (website)
I-A/I-E: Flow cytometric analysis of I-A/I-E expression on mouse splenocytes (website)
CD11b: Flow cytometric analysis of CD11b expression on mouse bone marrow (website)
Ly6G: Flow cytometric analysis of LY6G expression on mouse splenocytes (website)
CD4: Flow cytometric analysis of CD4 expression on mouse splenocytes (website)
CD8: Flow cytometric analysis of CD8 expression on mouse splenocytes (website)
CD3: Flow cytometric analysis of CD3 expression on mouse splenocytes (website)
CD19: Flow cytometric analysis of CD19 expression on mouse splenocytes (website)
F4/80 (clone BM8), Flow cytometric analysis of F4/80 expression on Thioglycolate-elicited BALB/c mouse peritoneal macrophages stained with BM8 APC (website).
Ly-6G: Flow cytometric analysis of Ly-6G expression on mouse bone marrow cells (website)
Ly-6C: Flow cytometric analysis of Ly-6 expression on mouse bone marrow cells (website)
CD69: Flow cytometric analysis of CD69 expression on mouse splenocytes (website)
CD25: Flow cytometric analysis of CD25 expression on mouse splenocytes (website)
CD62L: Flow cytometric analysis of CD62L expression on mouse bone marrow cells (website)
CD44: Flow cytometric analysis of CD 44 expression on mouse splenocytes (website)
CD86: Flow cytometric analysis of CD 86 expression on mouse splenocytes (website)
anti–PD-L1 for the in vivo treatment experiments in Fig4 (mouse IgG1 clone 6E11) - Genentech internal validation (in vitro and in vivo potency on MC38 and EMT6 subcutaneous tumors)
anti–TGF-β for the in vivo treatment experiments in Fig4 (mouse IgG1 clone 1D11) - Genentech internal validation (in vitro and in vivo

potency on MC38 and EMT6 subcutaneous tumors)

Eukaryotic cell lines

Policy information about cell lines and Sex and Gender in Research

Cell line source(s)	B16F10, EMT6, CT26, 4T1 mouse lines have been sourced from ATCC. KPP (PDAC) and NSCLC cells are derived from primary tumors of Genentech Cancer Immunology GEMM mice.
Authentication	Genentech built a centralized cell bank, gCELL, to support the needs of cell based research within Genentech. gCELL is tasked to bank verified, Quality Assured cell lines for distribution throughout Genentech. This provides a consistent source of cell lines for all levels of research to enable experimental reproducibility and access to baseline information such as morphology, growth conditions, RNAseq and ExomeSeq derived from these lines. gCELL also provides an important mechanism to ensure cell lines are used in accordance with all terms and conditions.
Mycoplasma contamination	Mycoplasma Testing. All stocks are tested for mycoplasma prior to and after cells are cryopreserved.<br>Two methods are used to avoid false positive/negative results: Lonza Mycoalert and Stratagene<br>Mycosensor. All cell lines tested negative for mycoplasma.
Commonly misidentified lines<br>(See ICLAC register)	N/A

Animals and other research organisms

Policy information about studies involving animals; ARRIVE guidelines recommended for reporting animal research, and Sex and Gender in Research

Laboratory animals	Male animals between 8–12 weeks old that appeared healthy and free of obvious abnormalities were used for the study. B6.Cg-Foxn1nu/J (Stock No. 000819), C57BL/6-Tg(CAG-EGFP)1Osb/J (Stock No. 003291), and C57BL/6J (Stock No. 000664) animals were purchased from the Jackson Laboratories (ME, USA). B6.129S6-Rag2tm1Fwa N12 (Model No. RAGN12), C.Cg/AnNTac-Foxn1nu NE9 (Model No. BALBNU-M), and BALB/cAnNTac (Model No. BALB-M) animals were purchased from Taconic Biosciences (CT, USA). CD4.cre.tg Rosa26.LSL.tdTomato.cki OT-I.TCR.tg (OT1-/- and OT1+/+) animals were bred in house and have been previously described. E8I.CD8A.IRES.GFP.Cre.tg Rosa26.LSL.tdTomato.cki animals were bred in house.<br>Animals were maintained in accordance with the Guide for the Care and Use of Laboratory Animals (National Research Council 2011). Genentech is an AAALAC-accredited facility and all animal activities in this research study were conducted under protocols approved by the Genentech Institutional Animal Care and Use Committee (IACUC).<br>Mice were housed in individually ventilated cages within animal rooms maintained on a 14:10-hour, light:dark cycle. Animal rooms were temperature and humidity-controlled, between 68 to 79°F (20.0 to 26.1 °C) and 30 to 70% respectively, with 10 to 15 room air exchanges per hour. For tumor inoculation studies: maximum total tumor volume before euthanasia is 2000 mm3.
Wild animals	No wild animals were used in this study
Reporting on sex	To keep results homogeneous, we used only male mice between 8–12 weeks old for this study. As tumor immunity is shared between males and females we believe the conclusions of this study apply to both females and males.
Field-collected samples	No field collected samples were used
Ethics oversight	Ethical approval for mouse experiments was obtained by the IACUC at Genentech (south San Francisco, CA)

Note that full information on the approval of the study protocol must also be provided in the manuscript.

Clinical data

Policy information about clinical studies

All manuscripts should comply with the ICMJE guidelines for publication of clinical research and a completed CONSORT checklist must be included with all submissions.

Clinical trial registration	IMvigor210 (previously published): NCT02951767/NCT02108652, ICON7: NCT00483782 (previously published) and imCORE trial (not published): NCT03333655
Study protocol	ICON7 and IMvigor210 studies have reported and the full protocols are available on clinicaltrials.gov.<br>For imCORE trial, protocol available upon request from www.roche.com/about_roche/roche_worldwide.htm +1 888-662-6728<br>global-roche-genentech-trials@gene.com
Data collection	This information is also available in previous publications or on clinicaltrials.gov for ICON7 and IMvigor210.<br>For imCORE trial, patients presented in this manuscript were recruited between January 2018 and March 2020. Data was generated from formalin-fixed parafin-embedded tumor blocks after biopsies were obtained.
Outcomes	Primary and secondary outcomes are all described on clinicaltrials.gov for ICON7 and IMvigor210. Not applicable for imCORE at that time.

Flow Cytometry

Plots

Confirm that:

☒ The axis labels state the marker and fluorochrome used (e.g. CD4-FITC).

☒ The axis scales are clearly visible. Include numbers along axes only for bottom left plot of group (a 'group' is an analysis of identical markers).

☒ All plots are contour plots with outliers or pseudocolor plots.

☒ A numerical value for number of cells or percentage (with statistics) is provided.

Methodology

Sample preparation	Single cell suspension was generated from mouse ear microtumors or subcutaneous tumors. Both tissues were digested with phosphate buffered saline containing 0.1 mg/mL DNAse I (Roche, Switzerland) and collagenase D at 1 mg/mL (Roche, Switzerland) for 30 min at 37°C to obtain a single cell suspension.
Instrument	BD LSRFortessa
Software	FACS Diva software version 7 (BD)
Cell population abundance	Abundance of each cell populations was assessed by flow cytometry
Gating strategy	A Gate based on fsc-a/ssc-a was set to include all cell populations, but excluding debris. 2. Gate on fsc-a vs. fsc-w was set to exclude doublets. 3. gate Fixable on the Viability Dye eFluor™ 780 was used to irreversibly label dead cells. Live singlets cells subsets CD45+ were gated as follow: MHC class II+, CD11c+, F4/80- as dendritic cells, MHC class II+, CD11c+, F4/80-CD103+ as dendritic cells type 1 subpopulation, MHC class II+, CD11c+, F4/80-, CD86+ as mature dendritic cells; CD11c-, CD11b+, Ly6G+, Ly6Clow as neutrophils; CD11c-, CD11b+, Ly6Glow, Ly6C+ as monocytes; CD11c-, CD11b+, F4/80+ as macrophages; CD3+ T cells were divided in CD4+ T cells and CD8+ T cells; CD8+ CD69+ activated/resident T cells, CD3+ CD44+ effector/ memory T cells and CD3+ CD62L+ naive T cells.

☒ Tick this box to confirm that a figure exemplifying the gating strategy is provided in the Supplementary Information.
